# Systems analysis of ethanol production in the genetically engineered cyanobacterium *Synechococcus* sp. PCC 7002

**DOI:** 10.1186/s13068-017-0741-0

**Published:** 2017-03-06

**Authors:** Joachim Kopka, Stefanie Schmidt, Frederik Dethloff, Nadin Pade, Susanne Berendt, Marco Schottkowski, Nico Martin, Ulf Dühring, Ekaterina Kuchmina, Heike Enke, Dan Kramer, Annegret Wilde, Martin Hagemann, Alexandra Friedrich

**Affiliations:** 1 Max-Planck-Institute of Molecular Plant Physiology, Am Mühlenberg 1, 14476 Potsdam-Golm, Germany; 2 Max-Planck-Institute of Psychiatry, Kraepelinstraße 2-10, 80804 Munich, Germany; 3 Institute of Biological Sciences, Plant Physiology, University of Rostock, Albert-Einstein-Str. 3, 18059 Rostock, Germany; 4 Algenol Biofuels Germany GmbH, Magnusstraße 11, 12489 Berlin, Germany; 5 Institute of Biology III, University of Freiburg, Schänzlestr. 1, 79104 Freiburg, Germany; 6 Cyano Biotech GmbH, Magnusstraße 11, 12489 Berlin, Germany

**Keywords:** *Synechococcus* sp. PCC 7002, Carbon assimilation, Carbon partitioning, Cyanobacteria, Ethanol, Glycolysis, Metabolomics, Proteomics, Pyruvate

## Abstract

**Background:**

Future sustainable energy production can be achieved using mass cultures of photoautotrophic microorganisms, which are engineered to synthesize valuable products directly from CO_2_ and sunlight. As cyanobacteria can be cultivated in large scale on non-arable land, these phototrophic bacteria have become attractive organisms for production of biofuels. *Synechococcus* sp. PCC 7002, one of the cyanobacterial model organisms, provides many attractive properties for biofuel production such as tolerance of seawater and high light intensities.

**Results:**

Here, we performed a systems analysis of an engineered ethanol-producing strain of the cyanobacterium *Synechococcus* sp. PCC 7002, which was grown in artificial seawater medium over 30 days applying a 12:12 h day–night cycle. Biosynthesis of ethanol resulted in a final accumulation of 0.25% (v/v) ethanol, including ethanol lost due to evaporation. The cultivation experiment revealed three production phases. The highest production rate was observed in the initial phase when cells were actively growing. In phase II growth of the producer strain stopped, but ethanol production rate was still high. Phase III was characterized by a decrease of both ethanol production and optical density of the culture. Metabolomics revealed that the carbon drain due to ethanol diffusion from the cell resulted in the expected reduction of pyruvate-based intermediates. Carbon-saving strategies successfully compensated the decrease of central intermediates of carbon metabolism during the first phase of fermentation. However, during long-term ethanol production the producer strain showed clear indications of intracellular carbon limitation. Despite the decreased levels of glycolytic and tricarboxylic acid cycle intermediates, soluble sugars and even glycogen accumulated in the producer strain. The changes in carbon assimilation patterns are partly supported by proteome analysis, which detected decreased levels of many enzymes and also revealed the stress phenotype of ethanol-producing cells. Strategies towards improved ethanol production are discussed.

**Conclusions:**

Systems analysis of ethanol production in *Synechococcus* sp. PCC 7002 revealed initial compensation followed by increasing metabolic limitation due to excessive carbon drain from primary metabolism.

**Electronic supplementary material:**

The online version of this article (doi:10.1186/s13068-017-0741-0) contains supplementary material, which is available to authorized users.

## Background

The identification of processes for sustainable production of energy is one of the global challenges to counteract effects of the increasing world population, of limited resources and of associated environmental problems. The use of photosynthetic organisms for energy and feedstock production represents one way to achieve nearly CO_2_-neutral processes. Oxygenic photosynthesis uses solar energy for the assimilation of CO_2_ into organic compounds. This process was “invented” about 2.5 billion years ago by ancient cyanobacteria and was later conveyed via endosymbiosis into eukaryotes giving rise to the development of diverse algal groups and land plants [1]. These photoautotrophic organisms are responsible for the production of most of the organic carbon and nitrogen to feed all heterotrophic organisms in the past as well as currently. Moreover, the fossil fuels, which presently dominate conventional energy production and lead to the rise in atmospheric CO_2_, also resulted from the accumulation of biomass of photoautotrophic organisms during ancient geological times.

Since photosynthesis efficiently converts solar energy into organic carbon that can be used for versatile purposes and needs only water and some inorganic nutrients, photoautotrophic organisms are increasingly applied for diverse biotechnological purposes. Initially, sugars produced by crop plants such as sugar cane were used for the fermentative production of ethanol by yeast, which is identified as a first-generation biofuel. However, this energy production directly competes with human nutrition with respect to biomass production and land use. Therefore, the alternative use of microalgae including cyanobacteria was suggested, since these organisms can be cultivated in large scale on non-arable land. In addition, cyanobacterial biofuel production can be combined with the reduction of CO_2_ in emissions from conventional power plants [2]. Moreover, freshwater is becoming a globally limiting resource. Therefore, the future mass cultivation of microalgae and cyanobacteria should be performed in seawater to conserve freshwater resources and to minimize the growth of competing organisms [3, 4].

Compared to microalgae, the biotechnological application of cyanobacteria offers many advantages since these prokaryotic organisms show high growth rates and many strains can be easily genetically modified. Ethanol-producing cyanobacterial strains were first generated by heterologous expression of the key enzymes pyruvate decarboxylase (PDC) and alcohol dehydrogenase (ADH) in the freshwater cyanobacterium *Synechococcus elongatus* PCC 7942 [5]. Subsequently, synthetic biology attempts were used to establish additional biosynthetic pathways in different cyanobacterial host cells, for example, to produce isoprene [6, 7], isobutanol [8], ethylene [9], lactate [10] and sucrose [11] among a plethora of additional biofuels and industrial products that can be produced under autotrophic conditions in cyanobacteria [2, 12, 13]. Recent systems biology analyses revealed that the implementation of biofuel synthetic pathways into the cyanobacterial cell may have greater impact on central metabolism and gene expression than initially expected. Metabolomics combined with transcriptomics showed that cells of *Synechocystis* sp. PCC 6803 (hereafter *Synechocystis* 6803), which were generated to produce isoprene, showed limitations in precursor synthesis and signs of redox-stress [7]. In contrast, prolonged ethanol production by *Synechocystis* 6803 had only minor impact on the transcriptome besides a gradual growth decline of producer cells [14].

In addition to implementing optimized synthesis genes and gene expression control elements, the host strain itself can have a major impact on product yield, especially under mass culture conditions. Previous attempts to produce biofuels at laboratory scales often used the freshwater model strains *S. elongatus* PCC 7942 (e.g. [5, 11, 15]) or *Synechocystis* 6803 (e.g. [7, 14, 16]). Another attractive cyanobacterial host for biotechnological purposes is the strain *Synechococcus* sp. PCC 7002 (hereafter *Synechococcus* 7002). *Synechococcus* 7002 is a euryhaline, unicellular cyanobacterium, which tolerates high light intensities and can grow in a wide range of NaCl concentrations [17–19]. Moreover, the strain is characterized by very fast growth rates (doubling times of 2.6–4.0 h; [20]). In addition, *Synechococcus* 7002 is also amenable to genetic modification [21] and several systems for genetic complementation and gene overexpression are available [22]. These features make *Synechococcus* 7002 a promising platform for biotechnological applications, including the production of biofuels [20].

Here we performed a systems analysis of an ethanol-producing strain of *Synechococcus* 7002, which was grown in artificial seawater medium for over 30 days. Ethanol accumulated continuously in the medium over more than 2 weeks. During prolonged fermentation periods, the producer strain gradually ceased growth and ethanol production. Metabolomics revealed that the carbon drain due to ethanol production resulted in reduction of pyruvate-based intermediates. During long-term ethanol production, the producer strain showed clear indications of intracellular carbon limitation. Despite the decreased levels of glycolytic and tricarboxylic acid cycle intermediates, soluble sugars and even glycogen accumulated in the producer strain. The main conclusions drawn from metabolome analysis on changes in carbon assimilation are supported by our proteome data. In the following, we will present data on the observed limitations on intracellular carbon allocation generated by ethanol synthesis, the compensation reactions of the ethanol-producing strain, and discuss strategies towards improving ethanol production by cyanobacteria.

## Results and discussion

### Ethanol production by engineered *Synechococcus* 7002 in seawater passes through three phases

**Fig. 1:**
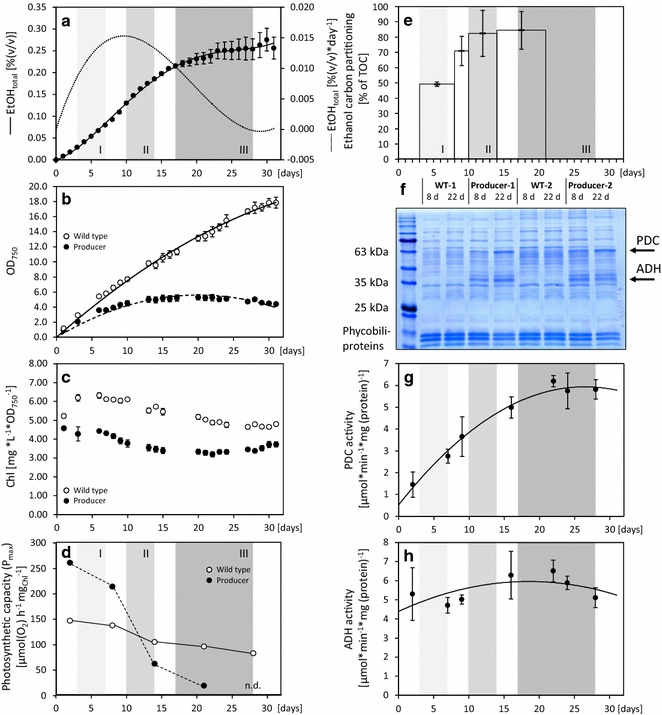
Cultivation and ethanol production data of a *Synechococcus* 7002 producer line compared to WT. **a** Vapor–liquid equilibria (VLE) corrected ethanol production and respective calculated rate of ethanol production per day (*dotted line*). *Trend lines* represent 4th and 3rd order polynomial regressions. **b** Optical density at wavelength *λ* = 750 (OD_750_). *Trend lines* represent 2nd order polynomial regressions. **c** Specific chlorophyll content calculated from chlorophyll and OD_750_ measurements (Additional file 1). **d** Maximum photosynthetic rate (*P*_max_) calculated as µmol (O_2_) h^−1^ mg_Chl_^−1^ of WT and the producer. Data were obtained from a representative cultivation (Additional file 1). **e** Carbon partitioning into ethanol (%) of the producer strain. Carbon partitioning was calculated from VLE corrected ethanol production relative to total organic carbon (TOC) in 4 cultivation intervals that span phases I–III as indicated. As TOC is calculated by subtracting measured total inorganic carbon (TIC) from measured total carbon (TC), it does not contain CO_2_ that is lost by enzymatic reactions, for example by pyruvate decarboxylation. Data calculated from two representative producer cultures. **f** Coomassie-stained SDS-PAGE of crude cell extracts (10 µg total protein, each lane) of WT and producer strain grown in parallel cultures at day 8 (at the transition from phase I to phase II) and day 22 (in the middle of phase III). Replicate 1 and 2 represent independent cultures. The ethanologenic gene cassette, consisting of *Zymomonas mobilis* pyruvate decarboxylase (PDC) and *Synechocystis* 6803 alcohol dehydrogenase (ADH), is located on the self-replicating plasmid #1449 (Additional file 9). Both proteins, the constitutively overexpressed ADH and the induced PDC are clearly present in the producer strain. Samples from the same cultures were additionally analyzed by blue-native PAGE (Additional file 10). **g** PDC activity in cell extracts of the ethanol producer. Data shown are averages of 4 replicates. **h** ADH activity in cell extracts of the ethanol producer. Data shown are averages of 4 replicates. Unless stated otherwise, data are averages ± standard deviation of 3–4 independent cultures from a 32 day batch cultivation (Additional file 1). Gray underlay indicates ethanol production phases I–III

An ethanol producer of *Synechococcus* 7002 expressing heterologously the pyruvate decarboxylase (PDC) gene from *Zymomonas mobilis* and the alcohol dehydrogenase (ADH) gene from *Synechocystis* 6803 was cultivated over 30 days in CO_2_-enriched seawater batch culture and was compared to the wild type (WT) grown under identical conditions. To avoid any potential effect on cellular physiology by antibiotic exposure, antibiotics were omitted from the growth media. Genetic stability of the ethanologenic gene cassette was indirectly monitored by measurements of PDC and ADH activities in cell extracts of cyanobacterial cultures. Ethanol production data, cell growth and non-targeted metabolome analyses revealed three phases of ethanol production of the *Synechococcus* 7002 producer strain (Fig. 1). The heterologous ADH gene was constitutively overexpressed from the genetically engineered Prbc*_(optRBS)_ promoter, whereas transcription of the heterologous PDC gene was induced on day 1. Therefore, synthesis of ethanol was initiated on day 1 of cultivation by PDC induction. Phase I coincided with early growth of the initial culture at 3–7 days post inoculation. During this phase the producer grew (as measured by change in optical density at 750 nm (OD_750_)) at a rate of 0.38 OD_750_ day^−1^. The ethanol production rate gradually increased during phase I and was on average 0.013% (v/v) ethanol day^−1^ (0.1 g L^−1^ day^−1^) as averaged across 4 photobioreactors (Fig. 1a; Additional file 1). These rates, that we report for the ethanol producer of *Synechococcus* 7002 in our 12:12 h (day–night) cultivation system with 230 μmol photons m^−2^ s^−1^, were only slightly less than half of the maximal rates that were reported for other genetically modified cyanobacteria grown under continuous light or much higher light intensities [12, 14] and were highly enhanced relative to the non-optimized initial *S. elongatus* PCC 7942 ethanol production system [5]. Phase II comprised the period 10–14 days post inoculation. In this phase growth of the *Synechococcus* 7002 producer strain stopped (Fig. 1b), but the rate of ethanol production remained high. Ethanol concentration still increased approximately linearly with time. The high ethanol production rate is caused by high amounts of heterologously expressed ADH and PDC (Fig. 1f–h). In phase III, 17–28 days post inoculation, growth remained stopped, and ethanol production of the producer line came to a standstill. PDC and ADH in vitro enzyme activities remained high throughout the entire cultivation (Fig. 1g, h), which strongly indicates a significant level of genetic stability of this ethanologenic gene cassette in *Synechococcus* 7002 for cultivation periods of at least 30 days. In contrast to the variable growth profile of the ethanol producer, *Synechococcus* 7002 WT maintained linear growth throughout the complete cultivation period. Suspensions of producer and WT cultivations differed in color. Chlorophyll content of the WT increased in phase I and declined in the further course of cultivation, whereas it did not increase in the producer strain and remained lower at 60–80% of the WT level (Fig. 1c). In addition, according to the Coomassie-stained SDS-PAGE (Fig. 1f) the amount of phycobiliproteins was reduced in the producer strain, especially in phase III after prolonged cultivation.

During phase I, the producer cells showed a significantly higher (about 1.5-fold) maximum photosynthetic rate (P_max_) than the WT (Fig. 1d; Additional file 1). Such a stimulation of photosynthesis has been already reported for cells of *S. elongatus* PCC 7942, which produced and excreted sucrose into the medium [11]. While the stimulation of photosynthesis was stable for many days in sucrose-producing cells [11], the photosynthetic activity of ethanol-producing cells of *Synechococcus* 7002 decreased quickly from this early maximum rate with ongoing cultivation (Fig. 1d). In phase II, the maximum photosynthetic rate of producer cells declined to ~60% of the WT level and became even lower during phase III. This decline in photosynthetic rate and the decrease in growth of producer cells likely resulted from ethanol production, causing an imbalance in primary carbon metabolism within the cell. It should be noted, however, that ethanol production is generated by high levels of expression of PDC and ADH (Fig. 1g, h). As such, a direct but non-specific impact of this level of protein overexpression and/or intracellular ethanol accumulation could have also had a negative effect on growth and photosynthetic rate. However, any effect on physiological properties (performance) caused by ethanol accumulation in the culture medium can be excluded. The final ethanol concentration in phase III without vapor–liquid equilibrium (VLE) correction was on average 0.19% (v/v) and never exceeded 0.23% (v/v) in the medium (Additional file 1). This concentration is far below the ethanol tolerance limit of *Synechococcus* 7002. *Synechococcus* 7002 is more tolerant against ethanol than the freshwater strains such as *Synechocystis* 6803 or *S. elongatus* PCC 7942 and has been shown to tolerate up to 0.2 M (approximately 1.16% v/v) ethanol in the medium without significant growth decline [23]. Ethanol tolerance of the producer strain was further supported by the finding that the overall partition of newly fixed organic carbon into ethanol increased from about 50% in phase I to almost 80% at the end of phase II and in phase III (Fig. 1e; Additional file 1). Thus, the relative carbon loss to ethanol relative to total organic carbon increased in parallel to decreased growth and photosynthetic activity of the producer.

**Fig. 2:**
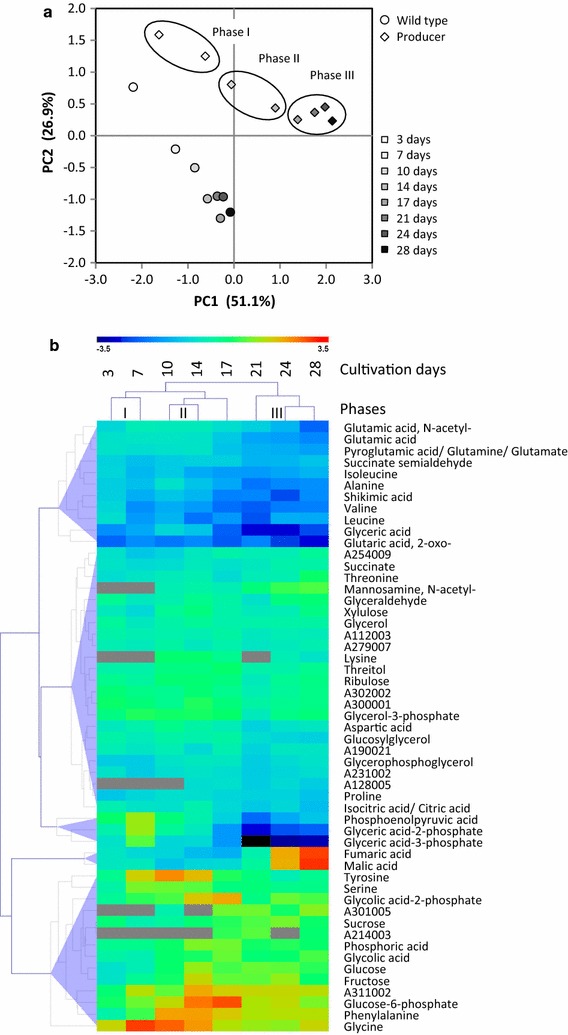
Global evaluation of metabolome analyses of the time course of ethanol production in an engineered *Synechococcus* 7002 producer strain compared to WT. **a** Principal component analysis (PCA) of log_2_-transformed metabolite pool size changes of producer and WT relative to the mean of each metabolite across all samples. Principal components, PC1 and PC2 represent 51.1 and 26.9% of total variance in the data set. **b** Hierarchical cluster analysis (HCA) of differential profiles, i.e. log_2_-transformed ratios of producer over WT at each time point. HCA was performed using Euclidian distance and average linkage. Data are averages of two producer and two WT batch cultures, respectively. Cells from each culture were harvested 6 times at each time point in the middle of a 12 h light phase (±10 min) as described under “Methods” section. WT and producer were probed in parallel, not sequentially. Cultivation phases I–III were assigned according to the matching PCA and HCA results

Consistent with the physiological data, in phase I metabolism of WT and the producer strain were overall quite similar but beginning to show distinct differences (Fig. 2a). Over the course of phases II and III, producer and WT metabolism became increasingly divergent. This trend is readily apparent through analysis of the differential metabolome profiles, i.e. log_2_-transformed ratios of producer over WT at each time point (Fig. 2b). Even though we differentiate three metabolic phases of ethanol production for the structured presentation of our results, it should be noted that these phases do not imply stepwise transitions between metabolic states. Instead, metabolic changes were continuous and in part overlapping. For example, glycolate-2-phosphate (2PG) and glucose-6-phosphate (Glc6P) transiently increased towards the end of phase II and at the beginning of phase III in a period 14–17 days post inoculation (Fig. 2b).

### The carbon drain from the pyruvate pool limits pools of the pyruvate amino acid family

**Fig. 3:**
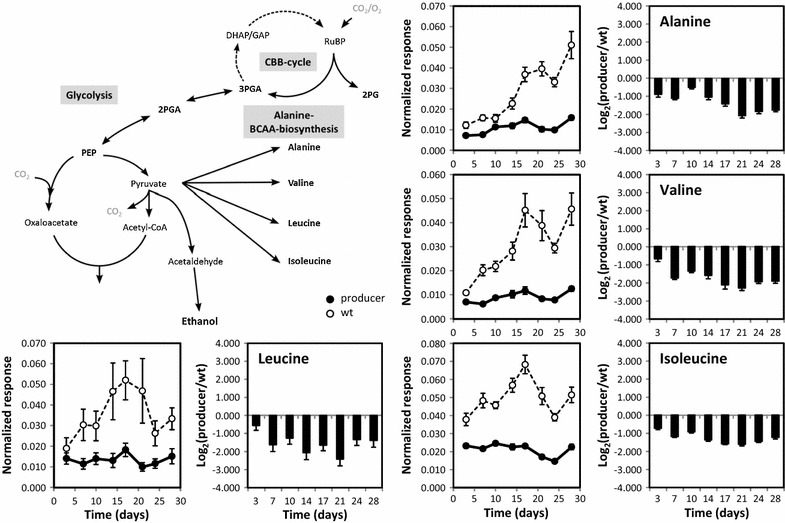
The scheme represents pyruvate dependent amino acid biosynthesis as well as engineered ethanol synthesis from pyruvate in *Synechococcus* 7002 expressing PDC and ADH. Metabolite data represent internal standard-corrected normalized responses, i.e. pool sizes in arbitrary units OD_750_^−1^ mL^−1^ of sample, from ethanol producer (*filled circles*) and WT (*open circles*). The *black columns* represent differential profiles, i.e. log_2_-transformed ratios of producer over WT at each time point (Additional file 3). Data are averages of two producer and two WT cultures, respectively. Cells from each culture were harvested six times at each time point in the middle of a 12-h light phase (±10 min) as described under “Methods” section

Ethanol production in *Synechococcus* 7002 diverts assimilated carbon from the pyruvate pool via acetaldehyde into ethanol. Between 50 and 80% of the organic carbon is irreversibly lost by diffusion of ethanol from the cell into the cultivation medium (Fig. 1e). This should decrease the pool size of pyruvate and all other pyruvate-based metabolites. Pyruvate was not detectable by routine non-targeted gas chromatography-electron impact ionization-mass spectrometry (GC/EI-TOF–MS) based metabolite profiling both in the WT and the producer strain, even by analysis of high biomass (approximately 15OD_750_ equivalents of cultivated cells). For this reason we established multi-targeted gas chromatography-atmospheric pressure chemical ionization-mass spectrometry (GC/APCI-qTOF-MS) analysis with enhanced sensitivity. However, pyruvate still remained below the detection threshold of the enhanced method. As a consequence, we used pyruvate-dependent pathways and respective metabolite pools as proxies of the effects of the carbon drain from the pyruvate pool during phase I and subsequent cultivation (Fig. 3).

The initial changes of primary metabolism in phase I are shown in Table 1. Metabolites with changed pool sizes are defined by *T* test significance *P* < 0.05 at day 3 and 7. Most of the metabolites with decreased pool sizes were either direct products of pyruvate, such as alanine and the branched chain amino acids isoleucine and valine, or dependent on carbon flux through the pyruvate pool and the 2-oxoglutarate (2OG) branch of the tricarboxylic acid (TCA) cycle, specifically glutamate, *N*-acetyl-glutamate, glutamine and pyroglutamate. Furthermore, succinate and succinate semialdehyde were significantly decreased in phase I. These metabolites are connected by the recently discovered alternative pathways, which close the cyanobacterial TCA cycle that lacks the 2OG dehydrogenase reaction [24–26]. In addition, the glycerate and glycerophosphoglycerol pools were decreased. Only the glycine pool and a yet non-identified metabolite were significantly increased in phase I (Table 1).

**Table 1:** Phase I changes of primary metabolism comparing the *Synechococcus* 7002 producer to WT

Metabolite	Response ratio (Producer/WT)		*T* test significance	
	Day 3	Day 7	Day 3	Day 7
	Log_2_	Log_2_	*P* < 0.05	*P* < 0.05
Alanine	−0.864	−1.083	1.0E−02	*8.3E−05*
Isoleucine	−0.725	−1.171	1.4E−03	*3.0E−05*
Valine	−0.677	−1.736	4.7E−03	*5.8E*−*05*
Glutamic acid	−0.506	−0.414	1.1E−02	*8.2E*−*05*
Glutamic acid, *N*-acetyl-	−0.681	−0.223	*2.8E*−*06*	1.9E−03
Glutamine (pyroglutamate, glutamate)^a^	−0.480	−0.544	6.7E−04	1.2E−04
Succinate	−0.435	−0.624	3.0E−03	*2.1E*−*06*
Succinate semialdehyde	−0.763	−1.075	*2.8E*−*07*	*9.8E*−*09*
Glyceric acid	−1.739	−1.410	*5.2E*−*05*	*5.7E*−*05*
Glycerophosphoglycerol	−0.860	−0.900	*1.2E*−*05*	*1.7E*−*07*
Glycine	1.741	2.954	*8.6E*−*07*	*1.1E*−*05*
A231002	−0.474	−0.868	2.0E−03	*7.8E*−*07*
A300001	0.603	0.468	3.2E−03	1.8E−02

Log_2_-transformed initial changes, i.e. response ratios of producer over wild type (WT), at low ethanol concentration (Fig. 1), were calculated and significance tested *P* < 0.05 using the *t* test (italics font: *P* < 0.0001)

^a^Pyroglutamic acid represents the sum of the glutamine, pyroglutamic and glutamic acid pool

Based on the observed reprogramming of primary metabolism of ethanol production in phase I, we hypothesized that an excessive carbon drain from the pyruvate pool interfered immediately with biosynthesis of alanine and branched-chain amino acids (BCAAs), which all directly require pyruvate as building blocks. This hypothesis was supported by continued and increased deficiency of these amino acids in producer cells during phases II–III (Fig. 3), whereas pools of alanine, valine, isoleucine, and in the later phases also of leucine significantly increased in WT cells over time. These observations indicate a continued limitation for the pyruvate family of amino acids in ethanol-producing *Synechococcus* 7002 cells (Fig. 3), consistent with the enhanced carbon partitioning into ethanol in phase III.

In addition, major parts of nitrogen metabolism were down-regulated, most probably due to limitation of carbon precursors. A detailed analysis of the 2OG branch of the TCA cycle showed that the producer strain became significantly deficient for 2OG and isocitrate/citrate throughout phases II-III (Additional file 2; note that the available analyte of this GC–MS based metabolite profiling study monitored the sum of the isocitrate and citrate pools). In comparison, the glutamate/glutamine/pyroglutamate deficiency was present throughout phase I–II and increased further in phase III (Additional file 2). Deficiency in nitrogen metabolism extended towards the proline and *N*-acetyl-glutamate pools (Additional file 3). This global decrease in N-assimilation could reflect a general cellular carbon-limitation that in turn diminishes the GS/GOGAT pathway for ammonia assimilation, as has been observed in cyanobacterial cells under inorganic carbon (Ci) limitation, i.e. after transfer from high to low CO_2_ conditions (e.g. [27–29]). Succinate semialdehyde levels behaved similar to the metabolites of the 2OG branch, i.e. its level was about twofold lower in the producer during the entire fermentation experiment (Additional file 2). This compound is synthesized from the precursors glutamate and 2OG in two alternative pathways closing the TCA cycle [24, 26], which also were diminished in producer cells. This consistent behavior indicates that the TCA cycle is less closed in ethanol-producing cells than in WT cells possibly due to the lowered overall carbon flux into the 2OG branch.

In contrast to glutamate, the aspartate pool remained unchanged in the producer strain compared to WT except towards the end of phase III (Additional file 4). This observation led us to the analysis of potential mechanisms that may compensate the effects of carbon drain from central metabolism. One mechanism that apparently contributes to aspartate homeostasis was the down-regulation of the C4-branch of the TCA cycle including the malate, fumarate and succinate pools in phases I–II (Additional file 4). We interpreted the depletion of the C4-branch of the TCA cycle as both recruitment of oxaloacetate for aspartate synthesis and of malate for pyruvate anaplerosis via decarboxylating malate dehydrogenase (malic enzyme) if pyruvate production from PEP by ATP producing pyruvate kinase becomes limiting. This anaplerotic reaction has been shown before to be the main route of newly fixed carbon via phosphoenolpyruvate (PEP) carboxylase in *Synechocystis* 6803 [30]. The compensation appeared to become deregulated (limited) and fails at the end of phase III, because then malate and fumarate increased strongly, 8.3- and 7.7-fold, respectively, whereas the succinate pool aligned to WT levels. This change may be related to the late mobilization of glycogen reserves in producer cells (discussed below).

Due to the decrease of the 2OG, glutamate and succinate semialdehyde pools, the C4-branch of the TCA cycle cannot be replenished by reactions that allow closure of the cyanobacterial TCA cycle via the γ-aminobutyrate (GABA) shunt or the 2OG decarboxylase path [24, 26]. The pool of succinate semialdehyde, which is a central intermediate of both pathways, was even more depleted than the succinate pool in the producer strain. This observation indicates that succinate is partly generated from fumarate in the C4-branch of the TCA cycle. Only in the WT, but not in the producer strain, was succinate semialdehyde highly correlated (Pearson’s correlation coefficient *r* > 0.750) with 2OG and glutamate/glutamine/pyroglutamate levels, but also with succinate pools (Additional file 5). These observations indicated that the TCA cycle was partly closed via succinate semialdehyde in the WT but was almost open in the producer strain due to the depletion of carbon flux into the TCA cycle.

### The carbon drain from the pyruvate pool is initially compensated but ultimately extends into the Calvin–Benson–Bassham (CBB) cycle

**Fig. 4:**
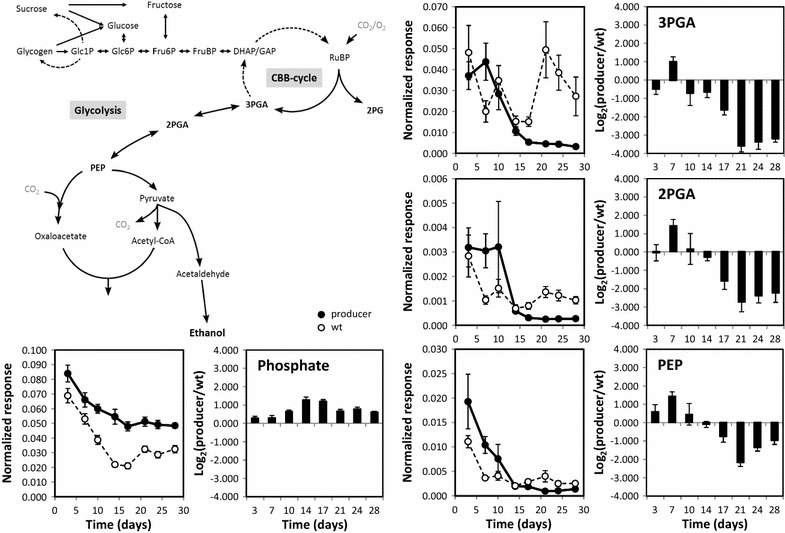
Scheme of glycolysis and major carbohydrate metabolism in relation to ethanol production from pyruvate in *Synechococcus* 7002. Metabolite data represent internal standard-corrected normalized responses, i.e. pool sizes in arbitrary units per OD_750_^−1^ mL^−1^ of sample, from ethanol producer and WT (*left*) and differential profiles (*right*), i.e. log_2_-transformed ratios of producer over WT at each time point (Additional file 3)

To further understand the homeostasis of the aspartate pool in the producer strain, we investigated the PEP pool and the upstream glycolytic pathway. Aspartate is generated by aspartate aminotransferase from oxaloacetate that in turn is the product of PEP carboxylase. This reaction bypasses the pyruvate pool and assimilates an additional carbon atom via carboxylation. While oxaloacetate like pyruvate was not detectable by our methods, PEP, 2-phosphoglycerate (2PGA) and 3-phosphoglycerate (3PGA) were accessible. The producer strain compensated and at day 7 of cultivation even over-compensated the carbon loss by ethanol production in phase I, since the pools of 3PGA and 2PGA were similar or in the case of PEP slightly increased compared to WT (Fig. 4). However, starting with phase II, all three pools became depleted. This observation was in agreement with the low flux rate through the pyruvate kinase reaction compared to the equally high fluxes through the enolase and phosphoglycerate mutase reactions, which were modeled for *Synechocystis* 6803 under photoautotropic conditions [30]. 3PGA, PEP and pyruvate levels are strongly depleted under photomixotrophic compared to photoautotrophic conditions [31]. Therefore, photomixotrophic conditions are difficult to compare to our study. However, the flux rate through the pyruvate kinase reaction also remains lower compared to the enolase flux under photomixotrophic [32] and autotrophic conditions [33]. The joined depletion of PEP, 2PGA and 3PGA in our study was accompanied by a consistent increase of intracellular inorganic phosphate indicating an overall loss of phosphorylated intermediates in the producer strain (Fig. 4). In phase III, specifically at day 21, 3PGA decreased ~tenfold relative to WT and was the most depleted compound among all monitored metabolite pools (Additional file 3). At this point the enhanced ethanol production in *Synechococcus* 7002 seems to cause an increasingly unbalanced carbon drain from central metabolism, which was initially compensated for. This drain is not met by sufficiently enhanced carbon assimilation rates because photosynthetic activity continuously declined after phase I (see Fig. 1d). As a consequence this imbalance first leads to a cessation of growth, and subsequently, of ethanol accumulation.

### The producer strain accumulates major carbohydrates

**Fig. 5:**
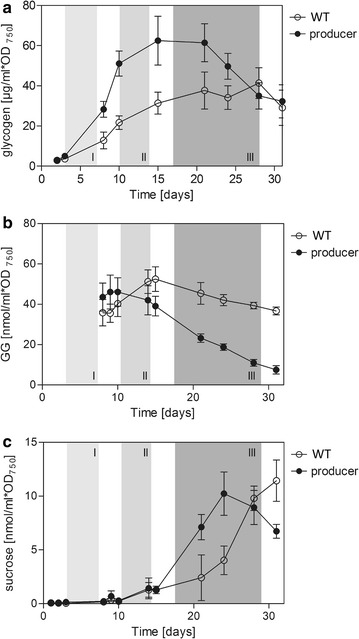
Amounts of glycogen (**a**), glucosylglycerol (GG) (**b**) and sucrose (**c**) in the ethanol producer compared to WT across the three phases (I–III) of ethanol production. Glycogen: µg mL^−1^ OD_750_^−1^ (mean values and standard deviation of two independent experiments with two technical repeats each). Glucosylglycerol: nmol mL^−1^ OD_750_^−1^ (mean values and standard deviation of two independent experiments with three technical repeats each). Sucrose: nmol mL^−1^ OD_750_^−1^ (mean values and standard deviation of two independent experiments with three technical repeats each)

Based on the observed progressive and joined depletion of the 3PGA, 2PGA and PEP pools we asked the question whether the major carbohydrate metabolism was equally affected by the carbon drain. An initial correlation analysis, however, indicated an inverse correlation of major carbohydrates to 3PGA in the WT (Table 2). This inverse correlation of glucose or fructose, for example, was still present in the producer strain but attenuated. In detail, we unexpectedly found increased glucose, fructose, sucrose and Glc6P levels from phase II onwards (Additional file 6). We verified sucrose accumulation in phase III by independent cultivation experiments for exact quantification of pool sizes and found in addition significant glycogen accumulation in the producer strain in phases II–III (Fig. 5). Glycogen serves as the main carbon storage molecule in cyanobacteria, which is typically accumulated in cells at excess carbon assimilation, for example, under conditions of nitrogen deprivation and iron deficiency. Glycogen is subsequently degraded under carbon-deficient conditions, such as dark periods to provide carbon precursors and energy [34, 35]. Contrary to our expectations, the *Synechococcus* 7002 producer strain did not efficiently mobilize glycogen and soluble carbohydrates for 3PGA anaplerosis and thus for the maintenance of the CBB cycle. The producer strain rather intensified the 3PGA deficiency by further withdrawing carbon for carbohydrate, specifically glycogen, accumulation. This behavior indicates that the ethanol-producing cells may not be generally limited by Ci assimilation but rather by the regulation of carbon allocation into different carbon pools. Specifically, a yet unknown regulatory mechanism linking major carbohydrate metabolism to the CBB cycle seems to be affected by ethanol production. This is in distinct contrast to CO_2_-limited cells of *S. elongatus* PCC 7942 and *Synechocystis* 6803 where we observed a coordinated decrease of CBB cycle intermediates, soluble carbohydrates and glycogen [29, 36].

**Table 2:** Correlation analysis of the variation of the 3PGA pool compared to all monitored metabolites of this study

Metabolite	Pearson’s correlation coefficient (*r*) of normalized responses		
	All samples	Wild type	Producer
Positive correlation			
Glyceric acid-3-phosphate	1.000	1.000	1.000
Glyceric acid-2-phosphate	0.806	0.767	0.450
Glutamic acid	0.821	0.755	0.557
Glutamine (pyroglutamic acid, glutamate)^a^	0.810	0.727	0.689
Glutamic acid, *N*-acetyl-	0.821	0.712	0.676
A112003	0.716	0.789	0.718
Negative correlation			
Mannose	0.408	−0.956	−0.615
Glucose	0.112	−0.774	−0.557
Fructose	0.068	−0.760	−0.640
Phenylalanine	0.011	−0.700	−0.642
A214003	0.224	−0.733	−0.718
A311002	−0.159	−0.732	−0.498

Pearson’s correlation coefficient of the WT subset were ranked and filtered by *r* > 0.70 or *r* < −0.70

Correlation coefficients of normalized responses from all, WT, or producer samples are reported. The complete data set can be found in Additional file 1

^a^Pyroglutamic acid represents the sum of the glutamine, pyroglutamic and glutamic acid pool

### The producer strain maintains high osmolyte concentrations in phases I and II

Because *Synechococcus* 7002 cultivation was performed in artificial seawater at 35 practical salinity units (PSU), we assumed that osmolyte biosynthesis presents a possibly competing mandatory carbon sink, since it is required for survival under the chosen culture conditions. *Synechococcus* 7002 accumulated the main compatible solute glucosylglycerol (GG) under our cultivation conditions to a level of 30–50 nmol mL^−1^ OD_750_^−1^ (Fig. 5; Additional file 3). GG is synthesized from glycerol-3-phosphate and ADP-glucose [4, 37]. The producer strain maintained almost WT levels of GG up to the end of phase II (Fig. 5). In parallel glycerol-3-phosphate levels were slightly increased relative to WT, whereas glycerol levels in the producer did not differ from WT (Additional file 3). However, in phase III the GG pool continuously decreased in producer cells, while it was kept constant in the WT (Fig. 5). At the same time, amounts of sucrose, fructose and glucose levels increased. We hypothesized that the *Synechococcus* 7002 producer strain used sucrose and possibly glucose and fructose as secondary osmolytes, when GG levels could not be maintained in phase III (Fig. 5; Additional file 6). But the total pool of osmotic active substances such as GG and soluble sugars was clearly lower than in WT cells during phase III. Thus, the requirement for osmolytes and survival may represent one reason why anaplerosis of the CBB cycle from soluble carbohydrate pools in the *Synechococcus* 7002 ethanol producing strain is not effective under seawater conditions. The observed imbalance in osmoregulation in the ethanol producer strain of *Synechococcus* 7002 could additionally contribute to the decrease in ethanol productivity, growth and maximum photosynthetic rate starting from phase II.

### The producer strain accumulates aromatic amino acids

Tyrosine and phenylalanine pools and to a smaller degree lysine were increased in phases II and III. In contrast, the shikimate pool was depleted throughout all phases even though the PEP pool was initially maintained or even increased (Additional file 7). Thus, the accumulation of aromatic amino acids was unlikely caused by de novo synthesis. As a consequence we hypothesized that the accumulation of aromatic amino acids was caused by compensatory protein degradation, which is used to refill the decreased amino acid pools (see Table 1; additional file 3). Proposed protein degradation is consistent with the decreased amount of phycobilisomes in the producer cells (Fig. 1f). We argue that aromatic amino acids are less readily catabolized than other amino acids and cannot rapidly be used for anaplerosis of central metabolism.

### The metabolome of the producer strain indicates increased photorespiration

Because the ethanol-producer strain showed increasing 3PGA deficiency, we asked the question of whether the photorespiratory C2-cylce was activated, which converts two molecules of the oxygenase product 2PG into one molecule of 3PGA [38]. 2PG was found at increased levels throughout all phases of ethanol production, whereas glycolate levels increased from phase II onwards (Additional file 8). In addition, the amino acid pools of glycine and serine increased significantly and peaked in phase I. These changes of photorespiratory C2-cycle intermediates did not extend into the glycerate pool which was depleted throughout (Additional file 3). Our previous analyses of acclimation processes during Ci limitation revealed the photorespiratory burst as central response in *Synechocystis* 6803. The photorespiratory burst characterizes a transient followed by a longer term adjustment of the 2PG, glycolate, glycine, serine pools, but not of glycerate [28, 39, 40] in CO_2_-limited WT cells. Similar changes were detected in photorespiratory mutants of *Synechocystis* 6803 that either lack essential enzymes of the photorespiratory C2-cycle [36, 41] or proteins to form carboxysomes [42]. These mutants already increased the pools of the photorespiratory C2-cycle intermediates under CO_2_-supplemented conditions. Here we found the similar accumulation of photorespiratory intermediates in the *Synechococcus* 7002 producer strain when cultivated under high CO_2_ conditions (10%, v/v). Therefore, we concluded that ethanol production induced increased photorespiration, which could be due to the decreased pools of specific organic carbon intermediates that may act in WT cells as signal molecule(s) for inducing the response to Ci limitation, or due to an increased permeability of carboxysomes for oxygen in the presence of cellular ethanol.

### Primary metabolism of ethanol production by enhanced *Synechococcus* 7002 partially phenocopies Ci limitation

Because enhanced ethanol production in the *Synechococcus* 7002 producer strain pointed in many aspects to intracellular carbon limitation, we performed a meta-analysis of the differential metabolome profiles of the producer over WT compared to Ci limitation of *Synechocystis* 6803 when shifted from high 5% CO_2_- to low 0.04% CO_2_-supply [36, 39]. We also included the data of a recently characterized *Synechocystis* 6803 mutant that can serve as model of engineered intracellular inorganic carbon (Ci) limitation, i.e., the *∆4* quadruple mutant (*∆ndhD3/ndhD4/cmpA/sbtA*-*∆sll1733/sll0027/slr0040/slr1512*), which lacks 4 of the 5 Ci-uptake systems [40, 43, 44]. This meta-analysis was enabled by the high CO_2_ cultivation system used in our study and by the fact that the previous studies of *Synechocystis* 6803 described the carbon limitation induced changes of metabolism relative to the respective high CO_2_-acclimated WT.

**Fig. 6:**
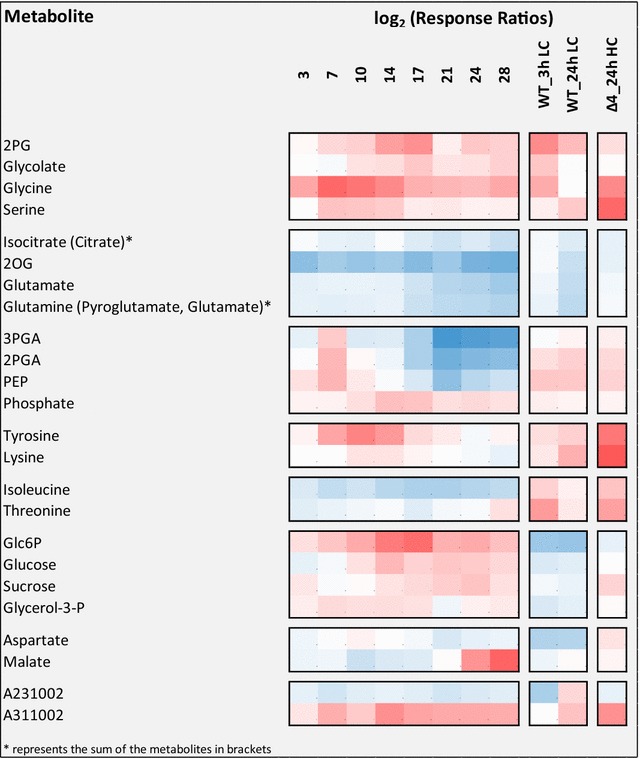
Meta-analysis of the differential profiles of the *Synechococcus* 7002 producer relative to WT compared to extracellular carbon limitation of the *Synechocystis* 6803 WT 3 h (WT_3 h LC) and 24 h (WT_24H LC) after shift from high (5%) to low (0.04%) CO_2_ supply [39, 40] and to intracellular carbon limitation under high CO_2_ conditions of the ∆*4* quadruple mutant of *Synechocystis* 6803 (∆*4_*24 h HC), which lacks 4 of the 5 Ci-uptake systems [40, 43, 44]. Note that all response ratios represent differential profiles of log_2_-transformed ratios calculated relative to the paired high CO_2_-acclimated WT of the respective study (Additional file 11)

The meta-analysis supported the view that many of the metabolic changes caused by ethanol production in *Synechococcus* 7002 mimic metabolic responses to Ci limitation that were previously observed in *Synechocystis* 6803 (Fig. 6). Specifically, the increase of photorespiratory intermediates, the parallel decrease of nitrogen metabolism and of TCA cycle intermediates as well as the increase of aromatic amino acids and lysine and finally the initial but transient compensatory increase of PEP, 2PGA and 3PGA all mirrored metabolic effects of Ci limitation. Differences were observed: (1) in pyruvate dependent branched-chain amino acids, which were depleted in the *Synechococcus* 7002 producer strain but increased by Ci limitation in *Synechocystis* 6803; (2) in the increased glycogen levels in the ethanol producing cells of *Synechococcus* 7002 compared to the lowered pool in Ci-limited *Synechocystis* 6803 cells (e.g. [36]); and (3) in the likely seawater cultivation dependent accumulation of soluble carbohydrates that decreased in freshwater cultivated *Synechocystis* 6803 (Fig. 6).

### Proteome analysis supports the notion of decreased carbon metabolism in the producer strain

The changes in the metabolome could result from changed enzyme abundances and/or regulation of enzyme activities at the post-translational level, for example via protein phosphorylation [45, 46]. For identification of changes in protein accumulation proteome analysis was performed using the 2-dimensional gel electrophoresis (2-DE) based method of protein separation with subsequent spot identification via mass spectroscopy (MS). This method does not represent the entire proteome, for example membrane proteins and proteins of low abundance are missing, whereas soluble proteins of the primary metabolism are well displayed (e.g. [47]). For analysis, sampling points at the transition from phase I to II (day 8) and at the transition into phase III (day 22) were selected. About 1700 spots were detected on the 2-DE gels. 192 spots showed potentially increased or decreased levels; however, 60% of those spots exhibited non-treatment specific variations, such as significant variations between replicates. Hence, only 75 spots showing reproducible treatment-related alterations were picked and the proteins identified. In this analysis we have focused our discussion on proteins with altered abundance (Table 3) that represent enzymes related to primary carbon metabolism or are involved in the cyanobacterial stress tolerance.

**Table 3:** Proteins with differential abundances in the ethanol producer compared to WT of *Synechococcus* 7002 at day 8 of the cultivation

ID cyano-base	Protein ID NCBI	Protein name	Gene name	Function (putative)	Fold change compared to WT (day 8)		
					Producer (day 8)	Producer (day 22)	WT (day 22)
Proteins with lower abundance in the producer strain compared to WT							
A0250	ACA98261	PEP synthase	*ppsA*	Glycolysis	0.77 (±0.24)	0.97 (±0.54)	2.75 (±0.21)
A0115	ACA98130	Glycosyl hydrolase		Sugar metabolism	0.02 (±0.028)	0.01 (±0.02)	1.49 (±0.13)
A1150	ACA99150	Glucosamine-fructose-6-phosphatase	*glmS*	Sugar metabolism	0.32 (±0.12)	0.16 (±0.06)	1.05 (±0.15)
A1172	ACA99171	1-Deoxy-D-xylulose-5-phosphate synthase	*dxs*	Isoprenoid synthesis	0.45 (±0.12)	0.53 (±0.17)	1.06 (±0.14)
A2851	ACB00819	GG-phosphate synthase	*ggpS*	Osmolyte synthesis	0.52 (±0.08)	0.09 (±0.02)	0.42 (±0.05)
A0330	ACA98340	4-Alpha-glucotransferase	*malQ*	Starch metabolism	0.18 (±0.07)	0.46 (±0.08)	1.26 (±0.16)
A2069	ACB00056	Adenosylhomocysteinase	*ahcY*	Amino acid synthesis	0.45 (±0.01)	0.47 (±0.06)	0.73 (±0.06)
A2819	ACB00788	Glycosyl hydrolase		Sugar metabolism	0.02 (±0.01)	0.07 (±0.08)	0.84 (±0.17)
A2665	ACB00642	Phosphoribulokinase	*prk*	Calvin cycle	0.33 (±0.12)	0.05 (±0.02)	0.66 9 (±0.26)
A0106	ACA98121	Glyceraldehyde-3-phosphate dehydrogenase	*gapI*	Calvin cycle	0.48 (±0.02)	0.46 (±0.04)	0.83 (±0.04)
A0353	ACA98363	Pyruvate dehydrogenase I		Pyruvate to acetylCoA	0.53 (±0.05)	0.35 (±0.03)	1.00 (±0.03)
A0452	ACA98460	Aldose 1-epimerase		Gluconeogenesis	0.33 (±0.13)	0.23 (±0.07)	1.43 (±0.09)
A1285	ACA99282	PII protein	*glnB*	Sensing cellular N	0.38 (±0.09)	0.29 (±0.09)	1.04 (±0.17)
A1549	ACA99540	Glycine dehydrogenase	*gcvP*	Glycine cleavage system P-protein	0.89 (±0.90)	0.42 (±0.18)	2.36 (±0.23)
Proteins with higher abundance in producer strain compared to WT							
A1321	ACA99318	Flavoprotein	*flv3*	Oxygen reducing flavodiiron protein	5.64 (±0.9)	6.33 (±0.14)	1.86 (±0.49)
A0605	ACA98612	Glycerol dehydrogenase	*gldA*	Glycerol metabolism	3.54 (±0.98)	14.94 (±5.50)	4.69 (±1.50)
A2458	ACB00436	Chaperonin 60 kDa	*groEL*	Heat shock protein	1.70 (±0.05)	1.10 (±0.29)	0.98 (±0.10)
A1803	ACA99791	Carbon concentrating mechanism protein	*ccmK*	–	2.33 (±0.30)	1.61 (±0.40)	1.1 (±0.10)
A2457	ACB00435	Chaperonin 10 kDa	*groES*	Heat shock protein	330.90 (±176.00)	219.13 (±270.00)	13.68 (±21.50)
A0147	ACA98161	Chaperonin Cpn60	*groEL II*	Heat shock protein	5.96 (±1.55)	3.08 (±1.20)	3.56 (±0.60)
A1800	ACA99788	Carbon concentrating mechanism protein	*ccmM*	–	1.73 (±0.27)	0.38 (±0.40)	0.16 (±0.30)
		Pyruvate decarboxylase	*pdc*	–	Present	Present	Absent

The fold changes to WT values on day 8 are given as fold change values ± standard deviation. Data present mean values and standard deviation of two independent cultures with two technical repeats each

Only few proteins showed increased abundances in producer cells compared to WT. As expected, the engineered PDC converting pyruvate into acetaldehyde was strongly over-expressed at protein level in the producer. Furthermore, the producer accumulated both, the core protein CcmM and the shell protein CcmK of carboxysomes [48] early and late in cultivation in comparison to WT (Table 3). This observation indicates that producer cells try to compensate the internal organic carbon limitation or carboxysome instability by an increased accumulation of carboxysomal proteins to maintain high photosynthetic CO_2_ assimilation. This interpretation is also supported by the metabolic data of phase I and the initially elevated maximum photosynthetic rate of the producer (Fig. 1d). The glycerol dehydrogenase was the only enzyme involved in primary carbon metabolism that increased in abundance in producer cells, which also accumulate higher levels of glycerol-phosphate. Interestingly, producer cells increased the abundance of several proteins that are involved in the general stress response of cyanobacterial cells. For example, the flavoprotein Flv3 (ACA99318) was highly up-regulated. This protein is known to catalyze the transfer of electrons from reduced ferredoxin towards O_2_ producing H_2_O in a Mehler-like reaction, which is assumed to function as energy dissipation system under high light or redox stress conditions [49–51]. In addition, the stress-induced chaperonins, GroEL, GroES and GroELII increased especially at the early time point. These proteins are involved in the assembly and repair of soluble proteins in the course of diverse stress treatments. For example, the GroEL and GroES chaperonins are induced by heat [52] and salt shock [47]. These observations may indicate that producer cells face a general stress situation possibly due to the imbalanced carbon metabolism. In addition, the lower cell density of the producer culture most probably enhances cell stress because of higher light intensity reaching these cells compared to WT (Fig. 1b). Similar results were found with cells of *Synechocystis* 6803 producing isoprene [7]. In contrast to our proteomic analysis, Qiao et al. [53] have examined changes in the proteome of *Synechocystis* 6803 in response to externally supplied ethanol. In this study, much higher concentrations of ethanol were used than the ethanol producer characterized in our study was able to accumulate. It is not surprising that there is only limited overlap between the two studies in terms of proteome response. For example, the heat shock protein GroES became elevated both by ethanol production as well as external ethanol application. In addition several metabolic proteins and surprisingly, photosynthesis proteins were found at decreased levels in response to ethanol, but only the amount of pyruvate dehydrogenase was also found to be decreased in our study.

Most of the observed and identified proteins show a lower abundance in the producer strain (Table 3). Among them are many enzymes involved in primary carbon metabolism. This finding is consistent with the observed intracellular carbon limitation. Specifically, the CBB enzymes phosphoribulokinase (Prk) and glyceraldehyde-3-phosphate dehydrogenase (GapI) are strongly downregulated at protein level in agreement with the metabolic depletion of 3PGA. In terms of pyruvate metabolism we found pyruvate dehydrogenase I with lower levels in the producer strain. This observation is consistent with a compensation reaction that potentially counteracts pyruvate depletion. Likewise, PEP synthase is not up-regulated at day 22 in the producer in comparison to WT. Again the missing activation of PEP synthesis from pyruvate is in agreement with pyruvate depletion.

Interestingly, the regulatory PII protein (GlnB), which has been identified as the central regulator of cyanobacterial nitrogen assimilation [54], also showed lower protein levels that are consistent with the generally decreased nitrogen metabolism found by our metabolome analysis. In addition, the GG-phosphatase synthase (GgpS) is lowered in the producer at both time points and thus reflects the lowered GG production. The P-protein subunit of glycine decarboxylase (GcvP) that is the active subunit for the glycine cleavage [55] is lowered in the producer contrary to expectations. Based on the metabolic indications of enhanced photorespiration one would expect intensified detoxification/recycling through the C2-cycle, but the glycine cleavage system protein P is decreased in the producer in comparison to WT. While the protein abundance of GcvP increased in WT from day 8 to day 22, this is not the case for the producer. This observation may explain why glycine remains highly increased in the producer.

## Conclusions

Our study revealed progressive intracellular organic carbon limitation caused by excessive carbon drain from central metabolism via ethanol loss. This conclusion is supported by the general trend of down-regulation of many associated enzymes. The pyruvate pool emerged as a likely limiting factor of ethanol production in *Synechococcus* 7002. The carbon drain was initially compensated for, but ultimately 3PGA depletion as a result of ethanol synthesis apparently interfered with efficient carbon assimilation through the CBB cycle and with the production of GG. GG, the main carbohydrate-based osmolyte of *Synechococcus* 7002, needs to be accumulated at a salinity-proportional level to maintain growth and cellular activity in seawater [37]. These metabolic imbalances transfer the producer cells into a stressful situation. Our systems analysis and the discovery of the three ethanol production phases lays the groundwork for future flux analyses experiments that require precise timing of the stable isotope pulse to unravel the paths of carbon utilization [33, 41]. Such experimental approaches and ^13^C metabolic flux analysis will allow identification of new design strategies for cyanobacterial metabolism [56, 57] and help to define limitations, such as the tradeoff between photosynthetic and metabolic robustness that apparently needs to be taken into account when engineering light-driven metabolic production processes [58]. On the basis of these observations, improvements in ethanol production could be accomplished by increasing the photosynthetic capacity of the cyanobacterial cell. Similar attempts have been reported, where the main carboxylating enzyme ribulose 1,5-bisphosphate carboxylase/oxygenase (RubisCO) has been over-expressed to improve the biofuel production with *S. elongatus* PCC 7942 [59]. Recent modeling studies suggested that adjustment of the relative intracellular ATP and NADPH concentrations according to the demands of biofuel production could substantially boost productivity [60]. Specifically, a lowered ATP to NADPH ratio than that generated by linear photosynthetic electron transport chain was predicted to increase ethanol yields. It is possible that this advantageous lower ratio and the anticipated increase in ethanol production could be achieved in vivo by moderately increasing ATP turnover through the addition of ATP wasting mechanisms, such as futile cycling [56]. In conclusion, a better understanding of the regulatory mechanisms of primary carbon metabolism that integrates ATP and NADPH cofactor demands will be helpful for the specific re-directing of newly assimilated carbon to rate-limiting or survival relevant metabolic pools.

## Methods

### Construction of the *Synechococcus* 7002 ethanol producer

Wild-type (WT) cells of *Synechococcus* 7002 were conjugated with the self-replicating plasmid #1449 (pVZ322a-*corR*-P*corT*-PDC-dsrA-Prbc*(optRBS)-*synADH*-oop). The ethanologenic plasmid #1449 contains the pyruvate decarboxylase gene from *Zymomonas mobilis* (zmPDC) under control of the Co^2+^ inducible promoter *PcorT* from *Synechocystis* 6803. *CorR*, encoding the transcriptional activator of *corT* was also cloned on plasmid #1449 (*corR sll0794* and *corT slr0797*). In addition, the ethanologenic gene cassette of the ethanol-producing strain #1449 contains the *Synechocystis* 6803 alcohol dehydrogenase (*slr1192*) under the transcriptional control of the genetically engineered P_rbc*(optRBS)_ promoter. The plasmid #1449 (Additional file 9) is based on pVZ322a, which is a derivative of pVZ321 [61]. Plasmid #1449 was generated by cloning the *corR*-P*corT*-zmPDC-TdsrA cassette into plasmid pVZ322a using *Sal*I/*Sbf*I sites for restriction and ligation. This cloning step removes the chloramphenicol resistance cassette (CmR) from the pVZ322a backbone and adds a unique *Spe*I restriction site between the TdsrA terminator and the SbfI site by insertion of the *corR*-P*corT*-zmPDC-TdsrA expression cassette. The Prbc*(optRBS)-synADH-Toop expression cassette was inserted subsequently via *Spe*I/*Sbf*I to form the final ethanologenic construct #1449 (Additional file 9).

### Cultivation conditions and sampling of *Synechococcus* 7002 ethanol producer and WT

WT and ethanol producer were cultivated in four replicates, each for 32 days, in 9-L vertical bioreactors. The bioreactor vessels are of 5 cm diameter. Cultures of WT and ethanol producer were grown under identical conditions in marine ASW (artificial seawater) medium, which is based on ASN-III medium [62] and adjusted to a salinity of 35 PSU (practical salinity unit). ASW is very similar to the cultivation medium used in Dienst et al. [14], where essentially BG11 medium [63] was supplemented with 35 g L^−1^ instant-ocean seawater salts (Aquarium Systems Inc, France) resulting also in 35 PSU. For growth of *Synechococcus* 7002 Vitamin B12 was added to the media (0.004 mg L^−1^). The cultivation vessel was fitted with ports for incoming pH- and temperature-sensors as well as sampling ports and connections to in-and out-gas. Cells were grown under day–night cycle conditions with a 12-h photoperiod. The light intensity was 230 μmol m^−2^ s^−1^ throughout this study. The culture temperature was controlled in a day–night cycle with a 37 °C daytime and 25 °C nighttime temperature. The cultures were continuously mixed by aeration, applying a gas flow rate of 250 mL min^−1^, to avoid settling of cells and the generation of sub-populations with different illumination. For the provision of sufficient CO_2_, the liquid phase was aerated with CO_2-_enriched air (10% CO_2_), by a pH-dependent and computer-controlled system. Induction of ethanol synthesis from the P_*corT*_ promoter in #1449 was triggered by adding 5 µM Co(NO_3_)_2_ to the reactor directly after inoculation on day 1 of cultivation.

### Monitoring of growth, chlorophyll content, ethanol production and total organic carbon (TOC)

Optical density (OD) was recorded at 750 nm 5 days per week. Chlorophyll contents were measured 3 times per week by spectrophotometry after extraction in 90% methanol [64]. Quantification of ethanol in the liquid phase was accomplished by head-space gas chromatography (GC) using a Shimadzu GC 2014 gas chromatograph as described by Dienst et al. [14]. To monitor ethanol production, the ethanol concentration in the liquid phase of the cultures was determined 5 days per week. In addition, the ethanol loss due to evaporation was calculated and experimentally verified. The ethanol concentration that is corrected for ethanol loss is called vapor–liquid equilibria (VLE) corrected ethanol. VLE-corrected and non-corrected data are given in Additional file 1.

The total carbon (TC) and the total inorganic carbon (TIC) were measured with a TOC analyzer TOC-VCPH equipped with an autosampler ASI-V and a combustion catalytic oxidation/non-dispersive infrared (NDIR) gas analyzer (Shimadzu Deutschland GmbH, Duisburg, Germany). The total organic carbon (TOC) was calculated using the differential method subtracting TIC from TC (TOC = TC − TIC). The calibration was made with commercially available standards (Bernd Kraft GmbH, Duisburg, Germany) and automatic dilution of standards using MilliQ water. The calibration range was from 5 to 500 mg C L^−1^ for TC and from 1 to 100 mg C L^−1^ for TIC. TOC was determined using whole culture volumes (cells plus medium). Samples were diluted with MilliQ water to a concentration of 20–180 mg C L^−1^ (estimated based on culture OD and ethanol content) in volumetric 50 mL flasks using volumetric pipettes. The diluted samples were transferred into 40 mL screw cap glass vials containing a magnetic stir bar (length: 1.2 cm) and sealed with a cap. For the measurement, a sample volume of 60 μL was injected into the TOC analyzer. Injections were repeated 3–5 times per measurement to obtain a mean value with a maximum standard deviation of 0.1.

### Assays to determine PDC and ADH activities in cell extracts of cyanobacterial cultures

For measurements of PDC and ADH activities in cell extracts of the ethanol producer strain, 1–20 mL cultures with a total OD_750_ of 10 were spun down (4500×*g*, 10 min at 4 °C). After cell lysis and centrifugation (13,000×*g*, 10 min at 4 °C), the supernatant was transferred into a fresh pre-chilled tube and the activities of PDC and ADH measured by opto-enzymatic assays.

To measure PDC activity a coupled enzyme assay was used: pyruvate (CAS 113-24-6, Sigma-Aldrich, Munich, Germany), added to the assay is converted in the presence of thiamine pyrophosphate (CAS 154-87-0, Sigma-Aldrich, Munich, Germany) by PDC in the cell extract of the ethanol producer to acetaldehyde. Acetaldehyde is then reduced to ethanol by ADH from *Saccharomyces cerevisiae*, added in excess to the reaction (CAS 9031-72-5, Sigma-Aldrich, Munich, Germany). The reaction consumes nicotinamide adenine dinucleotide (NADH). The PDC activity is determined by measuring the decrease in absorbance of NADH at 340 nm min^−1^.

To measure ADH, both, acetaldehyde and NADPH are added to the assay (CAS 75-07-0 and 2646-71-1, respectively, Carl Roth GmbH, Karlsruhe, Germany). Acetaldehyde is converted by the ADH in the cell extract of the producer to ethanol, oxidizing NADPH. The ADH activity is determined by measuring the decrease in absorbance of NADPH at 340 nm min^−1^. The specific activity of PDC and ADH was calculated by normalizing the activity to the total soluble protein concentration (mg mL^−1^) of the centrifuged lysate supernatant as determined by the method of Lowry et al. [65] and expressed as units of enzyme activity (µmol*min^−1^*mg (protein)^−1^).

### Photosynthetic measurements

Rates of photosynthetic oxygen evolution and oxygen consumption rates were measured with a Clark-type oxygen electrode (Rank Brothers, diameter 1 cm) at 37 °C. PI (photosynthesis irradiance) curves of the bicarbonate dependent photosynthetic oxygen evolution were measured for the WT and ethanol-producing culture at several days of cultivation. The cultures were diluted to the same amount of chlorophyll (2.5 µg) per mL in fresh medium supplemented with 5 mM NaHCO_3_. Light intensities were varied in the range 0–600 μmol photons s^−1^ m^−2^. The PE curves were analyzed according to Webb et al. [66] for *P*_max_ (oxygen evolution at saturating light intensities).

### Quantification of glucosylglycerol, sucrose and glycogen

Low molecular mass solutes were extracted from the cell pellets with 80% ethanol (HPLC grade, Roth, Germany) at 68 °C for 2 h. To quantify the internal amount of the molecular mass solutes, a defined amount of sorbitol was added to the samples as an internal standard. The extracts were centrifuged (13,000*g*, 5 min, 20 °C) and the supernatant was lyophilized. Dried pellets were suspended in distilled water and centrifuged (13,000*g*, 5 min, 20 °C) again. The supernatant was transferred to a new tube and lyophilized again. The final lyophilized extract was dissolved in pyridine, silylated and analyzed by gas chromatography (GC) according to Hagemann et al. [67].

Intracellular glycogen content was quantitatively measured using a colorimetric biochemical assay. Cells from 2 mL of culture were harvested and the pellet was re-suspended in 500 µL 80% ethanol (HPLC grade, Roth, Germany) and incubated for 2 h at 68 °C. The extracts were centrifuged (13,000*g*, 5 min, 20 °C) and the supernatant was used for the analysis of organic low molecular mass solutes, whereas the cell pellet was used for the glycogen determination. Then, the cell pellet was dried by lyophilization and re-suspended in 500 µL 30% (w/v) KOH. After incubation for 1 h at 100 °C the samples were cooled down and 625 µL of 95% (v/v) ethanol (HPLC grade, Roth, Germany) was added. The samples were again incubated at 100 °C for 15 min and afterwards centrifuged at 14,000 rpm for 10 min (20 °C). The resulting pellet was suspended in 500 µL deionized H_2_O at 100 °C for 15 min. After cooling on ice, 1 mL of freshly prepared anthrone reagent (200 mg anthrone in 100 mL 95% H_2_SO_4_) was added to the samples. The dye complex developed at 100 °C for 15 min, which was measured at 625 nm after a last cooling step. The resulting absorbance values were evaluated after comparison to a glycogen standard curve.

### Non-targeted metabolite profiling by gas chromatography-time of flight-mass spectrometry (GC/EI-TOF–MS)

6 technical replicates from two independent cultures of WT and producer, respectively, were harvested within less than 30 s until metabolic inactivation [36, 41] by fast filtration onto 25 mm diameter glass microfiber filters with 1.2 µm pore size (GE Healthcare, Little Chalfont, England), followed by immediate shock freezing [41]. Sampling by filtration was chosen instead of a freeze-quenching method because metabolic inactivation of cyanobacteria by freeze-quenching leads to a massive loss of intracellular metabolites during preparation [68]. Sampling was in the middle of the 12-h light phase over a period of 20 min. WT and producer cultures were sampled in parallel, not sequentially. Due to the changing OD in the course of cultivation, OD_750_ was measured at each sampling time before harvest and a respective volume was harvested (approx. 15 mL of an OD_750_ = 1.0 culture). Exact OD_750_ equivalents were recorded of each sample.

Metabolites were extracted from the deep frozen cells by a 2.5:1.0:1.0 (v/v/v) extraction mix containing methanol: chloroform: diethylamine (DEA; CAS 109-89-7, Merck Millipore, Darmstadt, Germany) and internal standards (ISTD). For this purpose 30 µL of an ISTD mixture was added to 1 mL of the extraction mix prior to extraction. The ISTD mixture was composed of 1 mg mL^−1^ ^13^C_5_^15^N-l-proline, 0.115 mg mL^−1^ ^13^C_12_-sucrose, 0.6 mg mL^−1^ ^13^C_3_^15^N-l-serine, 0.1 mg mL^−1 13^C_6_-d-glucose, 0.033 mg mL^−1^ ^13^C_6_-sorbitol, 0.1 mg mL^−1^ ^13^C_4_-succinat and 2 mg mL^−1^ ^13^C_6_-fructose-6-phosphate. Metabolites were extracted by adding 1 mL extraction volume, thorough mixing, 1 h incubation at 30 °C, induction of phase separation by adding 500 µL distilled water and retrieval of the upper polar fraction after 15 min centrifugation at 14,000 rpm using an Eppendorf 5417 centrifuge. An aliquot of 500 µL was dried in a 1.5-mL micro-centrifugation tube by vacuum centrifugation overnight and stored at −20 °C until further processing.

GC–MS based metabolite profiling including retention index (RI) standardization and chemical derivatization for GC analysis was performed as specified by Dethloff et al. [69]. One microlitre of standardized and chemically derivatized sample was injected onto a 30 m length, 0.25 mm inner diameter and 0.25 μm film thickness Varian FactorFour (VF-5 ms; Varian-Agilent Technologies, Waldbronn, Germany) capillary column coupled to electron impact ionization time-of-flight mass spectrometry (GC/EI-TOF–MS). GC/EI-TOF–MS was performed using an Agilent 6890N24 (Agilent Technologies, Waldbronn, Germany) gas chromatograph hyphenated to a LECO Pegasus III time-of-flight mass spectrometer as described [69]. Chromatograms were acquired and baseline corrected by ChromaTOF software (LECO Instrumente GmbH, Mönchengladbach, Germany). Metabolites were identified manually supervised using the TagFinder [70], the NIST08 software (http://chemdata.nist.gov/) and the mass spectral and retention time index (RI) reference collection of the Golm Metabolome Database [71].

### Enhanced targeted metabolite profiling by gas chromatography-atmospheric pressure ionization-quadrupole time of flight-mass spectrometry (GC/APCI-qTOF-MS)

Chemical derivatization and GC analysis were performed as described above. For RI calibration alkanes were replaced by a fatty acid methyl ester (FAME) mixture [72]. GC coupled to atmospheric pressure chemical ionization quadrupole time-of-flight mass spectrometry (GC/APCI-qTOF-MS) was performed using an Agilent 6890N24 gas chromatograph hyphenated to a Bruker impact II mass spectrometer (Bruker Daltonik GmbH, Bremen, Germany). Chromatograms were acquired with HyStar Version 3.2 software (Bruker Daltonik GmbH, Bremen, Germany) under control of the otofControl software Version 4.0.21.1960 (Bruker Daltonik GmbH, Bremen, Germany). Data mining was performed with Profile Analysis Version 2.2 software (Bruker Daltonik GmbH, Bremen, Germany). Line spectra were acquired in positive mode with scan rate 20 Hz and mass range 50–1500 *m/z*. Ion source parameters were: End plate offset 500 V, capillary 2000 V, corona current 3000 nA, nebulizer set to 3.5 bar (nitrogen), dry gas flow 2.5 L min^−1^ (nitrogen) and dry temp 250 °C. Tune parameters were: Transfer-Funnel 1 RF 300 Vpp, Funnel 1 RF 300 Vpp, in source CID energy 0.0 eV, hexapole Rf 60 Vpp, quadrupole-ion energy 4 eV, low mass cut-off 50 *m/z*, collision cell-collision energy 8 eV, collision RF 750 Vpp, transfer time 60 µs and pre-pulse storage 5 µs. External mass calibration was achieved by injection of 10 mM sodium formate solution, composed of 12.5 mL H_2_O, 12.5 mL isopropyl alcohol, 50 µL formic acid and 250 µL of 1 M NaOH in water. A single-syringe infusion pump 74,900 (Cole-Parmer Instrument Company, Vernon Hills, Illinois) was supplied with a Hamilton 1000-Series gastight 500 µL syringe (Hamilton, Bonaduz, Switzerland) set to a flow rate of 180 µL h^−1^.

For the purpose of multi-targeted datamining by Profile Analysis software, raw data analysis of positive mode line spectra was performed with spectral background subtraction and MS recalibration. MS recalibration setting were: Internal recalibration-calibration ESI, calibration list perfluortributylamin (PFTBA), Mode HPC, search range 0.01 *m/z*, intensity threshold 1000, retention time of the calibrant started at 0.07 min and ended at 0.08 min. So-called bucket generation parameters were: Ranges-retention time range start–end (min), mass range start–end (*m/z*); bucketing-rectangular bucketing delta (min) delta (*m/z*). The retention time (RT) range of each metabolite was defined by manual supervision with RT deviation set to ±0.5–1.0 min. The mass range of the expected molecular mass or fragment(s) was manually supervised expecting Gaussian shaped mass distributions and *m/z* deviation set to ±40 mDa. Analyte annotation was based on expected elution times calculated from Golm Metabolome Database retention indices using a FAME mixture [72]. In addition, the recorded APCI spectra were manually matched to EI reference spectra and to monoisotopic masses of expected protonated molecular ions and fragment ions. Ions used for relative quantification are listed in Additional file 3.

### Statistics and data visualization

Metabolite intensities were corrected for exact OD_750_ equivalents of each sample and normalized by ISTD intensity resulting in metabolite responses with units mL^−1^ OD_750_^−1^. ^13^C_6_-sorbitol was used as default internal standard except when metabolite-specific internal standards were available as listed in Additional file 3. Dimensionless response ratios were calculated comparing producer to WT at each time point of the cultivations and subsequently log_2_-transformed. Hierarchical cluster analysis with Pearson´s correlations distance and complete linkage and heat map visualizations were performed using the multi-experiment viewer software, MeV (Version 4.6.2; http://www.tm4.org/mev/; [73]). Principal component analysis (PCA) was performed using log_2_-transformed response ratios via the MetaGeneAlyse web application (Version 1.7.1; http://metagenealyse.mpimp-golm.mpg.de) with missing value substitution as was described earlier [74].

### SDS- and blue native (BN)-PAGE analysis

Whole-cell extracts of *Synechococcus* 7002 were prepared as described previously [75] and proteins were separated by SDS-PAGE [76] or BN-PAGE using precast NativePAGE Novex 4–16% Bis–Tris gels (Thermofisher, Germany). Preparation of thylakoid membranes for BN-PAGE analysis (Additional file 10) and gel electrophoresis was performed according to Lassen et al. [77].

### Proteomic analysis

#### Preparation of protein extracts preparation for proteomics

Cells corresponding to 120-150 OD_750_-units were spun down and the resulting cell pellets weighed. After resuspension in PBS buffer (140 mM NaCl; 2.5 mM KCl; 10 mM Na_2_HPO_4_; 2 mM KH_2_PO_4_, pH 7.4) suspensions containing 80–120 mg cell material were transferred to a new 2 mL tube and washed twice with PBS. Finally, the pellet was re-suspended in PF-Buffer (6 M urea; 3 M thiourea; 70 mM DTT; 12.5 mM Tris, pH 7.5 including the cOmplete Protease Inhibitor Cocktail, Sigma-Aldrich, St. Louis, Missouri, USA). 800 µL glass beads (size 0.1–0.25 mm) were added to the samples and cells broken by bead-beating. Afterwards unbroken cells and cell debris were pelleted by a 3-min spin with 6000 rpm at 4 °C. The supernatant fraction was collected in a 15-mL tube and kept on ice. To the remaining pellet 1 mL of PF buffer was added and another cycle of bead-beating was performed. After another 3 min spin with 2500 rpm both supernatants were combined and a last bead-beating of the pellet with subsequent centrifugation for 1 min with 5000 rpm was conducted. Finally, the collected supernatant fraction was sonicated for 5 × 10 s at 50% with incubation on ice in between cycles. At last, remaining debris was pelleted after 2 min at 3000 rpm, the supernatant was collected and stored at −20 °C overnight. To solubilize proteins from membranes Triton X-100 (end concentration 0.5%) was added to the slowly thawed sample and incubated at room temperature on a shaker for 30 min. Debris was pelleted during a 30-min spin at 13,000 rpm at 4 °C, and the resulting supernatant was digested with *Benzonase*® Nuclease (Sigma-Aldrich, St. Louis, Missouri, USA; 250 units/µL) in a 1:8000 dilution for 15 min at room temperature. After that, using Amicon Ultra Filter 10 K (Merck-Millipore, Darmstadt, Germany) samples were concentrated to an approximate end volume of 600 µL. Finally, after performing a precipitation with 10% trichloroacetic acid overnight at 4 °C with two subsequent washing steps with acetone, the pellet was re-suspended in PF buffer and used for analysis via two-dimensional electrophoresis.

#### Two-dimensional electrophoresis (2-DE)

2-DE was performed by the Proteome Factory AG (Berlin, Germany) based on the 2-DE technique according to Klose and Kobalz [78]. For the analysis 100 µg protein was mixed with 2% ampholytes (pH 2–11) and applied to vertical rod gels (9 M urea, 4% acrylamide, 0.3% piperazine diacrylamide (PDA), 5% glycerol, 0.06% N,N,N′,N′-tetramethylethylendiamin (TEMED) and 4% carrier ampholytes (pH 2–11), 0.02% ammonium persulfate (APS) for isoelectric focusing (IEF) at 8820 Vh in the first dimension. After focusing, the IEF gels were incubated in equilibration buffer, containing 125 mM trisphosphate (pH 6.8), 40% glycerol, 65 mM DTT, and 3% SDS for 10 min and subsequently frozen at −80 °C. The second dimension SDS-PAGE gels (23 cm × 30 cm × 0.1 cm) were prepared, containing 375 mM Tris–HCl buffer (pH 8.8), 15% acrylamide, 0.2% bisacrylamide, 0.1% SDS and 0.03% TEMED. After thawing, the equilibrated IEF gels were immediately applied to SDS-PAGE gels. Electrophoresis was performed using 140 mA for 5.5 h until the front reached the end of the gel. After 2-DE separation the gels were stained with FireSilver (Proteome Factory, PS2001). In total four 2D-gels were performed per strain and sampling time point, resulting from two independent cultures and two technical replicates each. Analyses of the 2-DE gels were performed according to Bernhardt et al. [79].

#### Protein identification by liquid chromatography-mass spectrometry

Protein identification using nanoLC-ESI–MS/MS was performed by Proteome Factory (Proteome Factory AG, Berlin, Germany). The MS system consisted of an Agilent 1100 nanoLC system (Agilent, Waldbronn, Germany), PicoTip electrospray emitter (New Objective, Woburn, USA, MA) and an Orbitrap XL or LTQ-FT Ultra mass spectrometer (ThermoFisher, Bremen, Germany). Protein spots were in-gel digested by trypsin (Promega, Mannheim, Germany) and applied to nanoLC-ESI–MS/MS. Peptides were trapped and desalted on the enrichment column (Zorbax SB C18, 0.3 × 5 mm, Agilent) for 5 min using 2.5% acetonitrile/0.5% formic acid as eluent, then peptides were separated on a Zorbax 300 SB C18, 75 µm × 150 mm column (Agilent) using an acetonitrile/0.1% formic acid gradient from 5 to 35% acetonitrile within 40 min. MS/MS spectra were recorded data-dependently by the mass spectrometer according to manufacturer’s recommendations. Proteins were identified using MS/MS ion search of the Mascot search engine (Matrix Science, London, England) and the protein database (National Center for Biotechnology Information, Bethesda, USA) Cyano_SP7002 (18,695 sequences). Ion charge in search parameters for ions from ESI–MS/MS data acquisition were set to “1+, 2+ or 3+” according to the instrument’s and method’s common charge state distribution. Further search parameters were the following: Peptide Mass Tolerance: ±5 ppm; Fragment Mass Tolerance: ±0.6 Da; Max Missed Cleavages: 1. The Mascot search results were filtered by selecting a *P* value <0.05.

## Additional files

**Additional file 1.** Ethanol production, optical density, chlorophyll content, ethanol carbon partitioning and maximum photosynthetic rate of the *Synechococcus* 7002 producer line compared to WT.

**Additional file 2.** Metabolites of nitrogen metabolism and of the 2-oxoglutarate (2OG) branch of the TCA cycle. Metabolite data represent internal standard-corrected normalized responses, i.e. pool sizes in arbitrary units OD_750_^−1^ mL^−1^ of sample, from ethanol producer and WT (left) and differential profiles (right), i.e. log_2_-transformed ratios of producer over WT at each time point (Additional file 3).

**Additional file 3.** Metabolite profiling analyses of the *Synechococcus* 7002 producer line compared to WT.

**Additional file 4.** Metabolites of the C4-branch of the TCA cycle. Metabolite data represent internal standard-corrected normalized responses, i.e. pool sizes in arbitrary units OD_750_^−1^ mL^−1^ of sample, from ethanol producer and WT (left) and differential profiles (right), i.e. log_2_-transformed ratios of producer over WT at each time point (Additional file 3).

**Additional file 5.** Correlation analysis of the variation of the succinate semialdehyde pool compared to all monitored metabolites of this study. Pearson´s correlation coefficient of the WT subset were filtered by r > 0.70 or r < -0.70. Correlation coefficients of normalized responses from all, WT, or producer samples are reported (the complete data set can be found in Additional file 3).

**Additional file 6.** Metabolites of major carbohydrate metabolism. Metabolite data represent internal standard-corrected normalized responses, i.e. pool sizes in arbitrary units OD_750_^−1^ mL^−1^ of sample, from ethanol producer and WT (left) and differential profiles (right), i.e. log_2_-transformed ratios of producer over WT at each time point (Additional file 3).

**Additional file 7.** Aromatic amino acids and shikimate. Metabolite data represent internal standard-corrected normalized responses, i.e. pool sizes in arbitrary units per OD_750_^−1^ mL^−1^ of sample, from ethanol producer and WT (left) and differential profiles (right), i.e. log_2_-transformed ratios of producer over WT at each time point (Additional file 3).

**Additional file 8.** The photorespiratory C2-cycle. Metabolite data represent internal standard-corrected normalized responses, i.e. pool sizes in arbitrary units per OD_750_^−1^ mL^−1^ of sample, from ethanol producer and WT (left) and differential profiles (right), i.e. log_2_-transformed ratios of producer over WT at each time point (Additional file 3).

**Additional file 9.** Plasmid maps of pVZ322a and its ethanologenic version #1449 (pVZ322a-corR-PcorT-PDC-dsrA-Prbc*(optRBS)-synADH-oop). The ethanologenic gene cassette contains the PDC gene from *Z.mobilis* (zmPDC) and the ADH gene from *Synechocystis* 6803. While expression of PDC is driven by the Co^2+^ inducible P*corT* promoter from *Synechocystis* 6803, ADH expression is under the control of the genetically engineered Prbc*_(optRBS)_ promoter. *corR* (*sll0794*) encodes the transcriptional activator of *corT* (*slr0797*).

**Additional file 10.** Blue-Native PAGE of protein samples from WT and producer, collected on day 8 and day 22 of the cultivation experiment. Thylakoid membranes equivalent to 8 µg of chlorophyll were separated on precast Novex 4-16% Blue native Gel and stained with Coomassie. Phycobiliproteins are reduced in the producer strain after prolonged cultivation.

**Additional file 11.** Meta-analysis of the differential profiles of the *Synechococcus* 7002 producer relative to WT at day 3-28 compared to extracellular and intracellular carbon limitation of *Synechocystis* 6803 (data extracted from [39, 40]).

## Abbreviations

ADH: alcohol dehydrogenase
CBB: Calvin–Benson–Bassham
Chl: chlorophyll
2-DE: two-dimensional gel electrophoresis
DMAPP: dimethylallyl diphosphate
DXS: 1-deoxy-d-xylulose 5-phosphate synthase
GC: gas chromatography
Glc6P: glucose-6-phosphate
GG: glucosylglycerol
MEP: 2-*C*-methyl-d-erythritol 4-phosphate pathway
OD: optical density
2OG: 2-oxoglutarate
OPP: oxidative pentose phosphate
PDC: pyruvate decarboxylase
PEP: phosphoenolpyruvate
2PG: glycolate-2-phosphate
2PGA/3PGA: 2/3-phosphoglycerate
TCA: tricarboxylic acid
TOC: total organic carbon
WT: wild type
