# Supplementary material for: Systems analysis of ethanol production in the genetically engineered cyanobacterium Synechococcus sp. PCC 7002
Source: Biotechnol Biofuels. 2017 Mar 6;10:56. doi: 10.1186/s13068-017-0741-0 (PMC5340023; doi:10.1186/s13068-017-0741-0)
Supplement: Supplementary file 2 — Additional file 2. Metabolites of nitrogen metabolism and of the 2-oxoglutarate (2OG) branch of the TCA cycle. Metabolite data represent internal standard-corrected normalized responses, i.e. pool sizes in arbitrary units OD750−1 mL−1 of sample, from ethanol producer and WT (left) and differential profiles (right), i.e. log2-transformed ratios of producer over WT at each time point (Additional file 3). [file 13068_2017_741_MOESM2_ESM.pptx]

## Slide 1
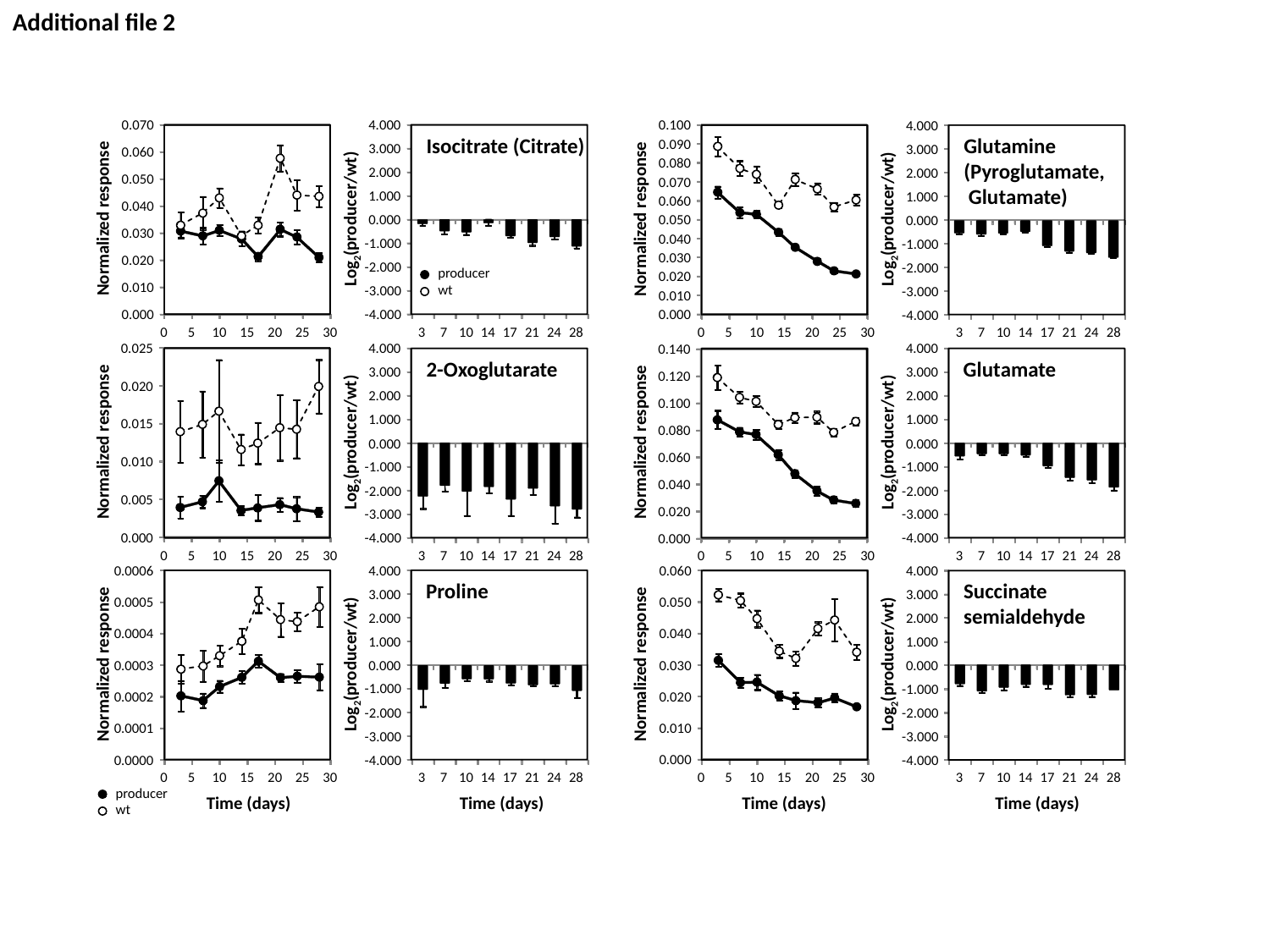

Additional file 2
4.000
0.070
Isocitrate (Citrate)
3.000
0.060
2.000
0.050
1.000
0.040
Normalized response
Log2(producer/wt)
0.000
0.030
-1.000
0.020
-2.000
0.010
-3.000
-4.000
0.000
0
5
10
15
20
25
30
3
7
10
14
17
21
24
28
0.100
4.000
Glutamine
(Pyroglutamate,
 Glutamate)
0.090
3.000
0.080
2.000
0.070
1.000
0.060
Normalized response
Log2(producer/wt)
0.050
0.000
0.040
-1.000
0.030
-2.000
0.020
-3.000
0.010
0.000
-4.000
0
5
10
15
20
25
30
3
7
10
14
17
21
24
28
producer
wt
0.025
4.000
2-Oxoglutarate
3.000
0.020
2.000
1.000
0.015
Normalized response
Log2(producer/wt)
0.000
0.010
-1.000
-2.000
0.005
-3.000
0.000
-4.000
0
5
10
15
20
25
30
3
7
10
14
17
21
24
28
4.000
0.140
Glutamate
3.000
0.120
2.000
0.100
1.000
0.080
Normalized response
Log2(producer/wt)
0.000
0.060
-1.000
0.040
-2.000
0.020
-3.000
-4.000
0.000
0
5
10
15
20
25
30
3
7
10
14
17
21
24
28
4.000
0.0006
Proline
3.000
0.0005
2.000
0.0004
1.000
Normalized response
Log2(producer/wt)
0.000
0.0003
-1.000
0.0002
-2.000
0.0001
-3.000
-4.000
0.0000
0
5
10
15
20
25
30
3
7
10
14
17
21
24
28
0.060
0.050
0.040
0.030
0.020
0.010
0.000
4.000
Succinate
semialdehyde
3.000
2.000
1.000
Normalized response
Log2(producer/wt)
0.000
-1.000
-2.000
-3.000
-4.000
0
5
10
15
20
25
30
3
7
10
14
17
21
24
28
producer
wt
Time (days)
Time (days)
Time (days)
Time (days)
