# Supplementary material for: Systems analysis of ethanol production in the genetically engineered cyanobacterium Synechococcus sp. PCC 7002
Source: Biotechnol Biofuels. 2017 Mar 6;10:56. doi: 10.1186/s13068-017-0741-0 (PMC5340023; doi:10.1186/s13068-017-0741-0)
Supplement: Supplementary file 4 — Additional file 4. Metabolites of the C4-branch of the TCA cycle. Metabolite data represent internal standard-corrected normalized responses, i.e. pool sizes in arbitrary units OD750−1 mL−1 of sample, from ethanol producer and WT (left) and differential profiles (right), i.e. log2-transformed ratios of producer over WT at each time point (Additional file 3). [file 13068_2017_741_MOESM4_ESM.pptx]

## Slide 1
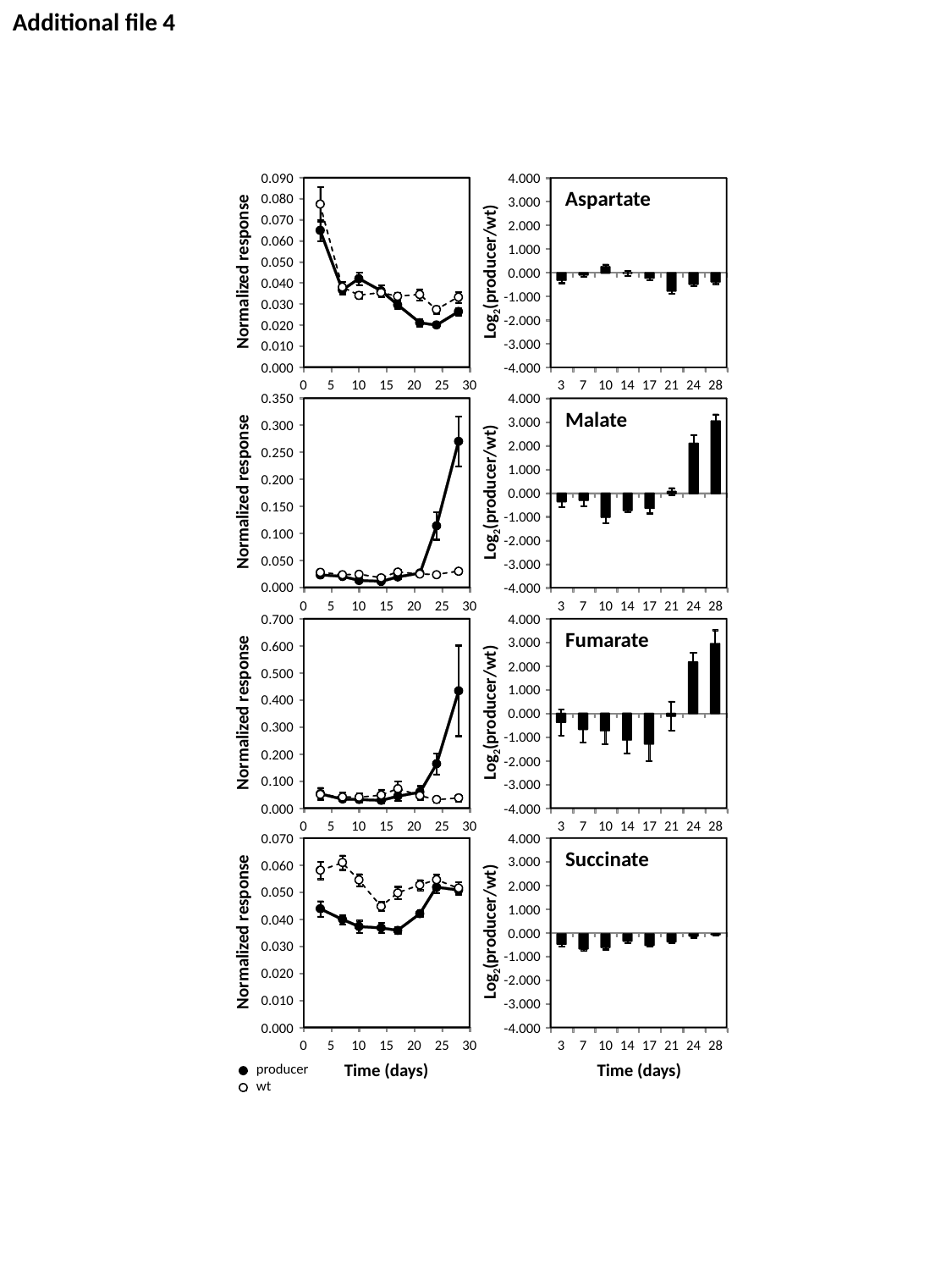

Additional file 4
0.090
0.080
0.070
0.060
0.050
0.040
0.030
0.020
0.010
0.000
4.000
Aspartate
3.000
2.000
1.000
Normalized response
Log2(producer/wt)
0.000
-1.000
-2.000
-3.000
-4.000
0
5
10
15
20
25
30
3
7
10
14
17
21
24
28
0.350
4.000
Malate
3.000
0.300
2.000
0.250
1.000
0.200
Normalized response
Log2(producer/wt)
0.000
0.150
-1.000
0.100
-2.000
0.050
-3.000
0.000
-4.000
0
5
10
15
20
25
30
3
7
10
14
17
21
24
28
4.000
0.700
Fumarate
3.000
0.600
2.000
0.500
1.000
0.400
Normalized response
Log2(producer/wt)
0.000
0.300
-1.000
0.200
-2.000
0.100
-3.000
-4.000
0.000
0
5
10
15
20
25
30
3
7
10
14
17
21
24
28
4.000
0.070
Succinate
3.000
0.060
2.000
0.050
1.000
0.040
Normalized response
Log2(producer/wt)
0.000
0.030
-1.000
0.020
-2.000
0.010
-3.000
0.000
-4.000
0
5
10
15
20
25
30
3
7
10
14
17
21
24
28
Time (days)
Time (days)
producer
wt
