# Supplementary material for: Systems analysis of ethanol production in the genetically engineered cyanobacterium Synechococcus sp. PCC 7002
Source: Biotechnol Biofuels. 2017 Mar 6;10:56. doi: 10.1186/s13068-017-0741-0 (PMC5340023; doi:10.1186/s13068-017-0741-0)
Supplement: Supplementary file 7 — Additional file 7. Aromatic amino acids and shikimate. Metabolite data represent internal standard-corrected normalized responses, i.e. pool sizes in arbitrary units per OD750−1 mL−1 of sample, from ethanol producer and WT (left) and differential profiles (right), i.e. log2-transformed ratios of producer over WT at each time point (Additional file 3). [file 13068_2017_741_MOESM7_ESM.docx]

**Additional file 7.** Aromatic amino acids and shikimate.
