# Supplementary figures and images for: Systems analysis of ethanol production in the genetically engineered cyanobacterium Synechococcus sp. PCC 7002
Source: Biotechnol Biofuels. 2017 Mar 6;10:56. doi: 10.1186/s13068-017-0741-0 (PMC5340023; doi:10.1186/s13068-017-0741-0)

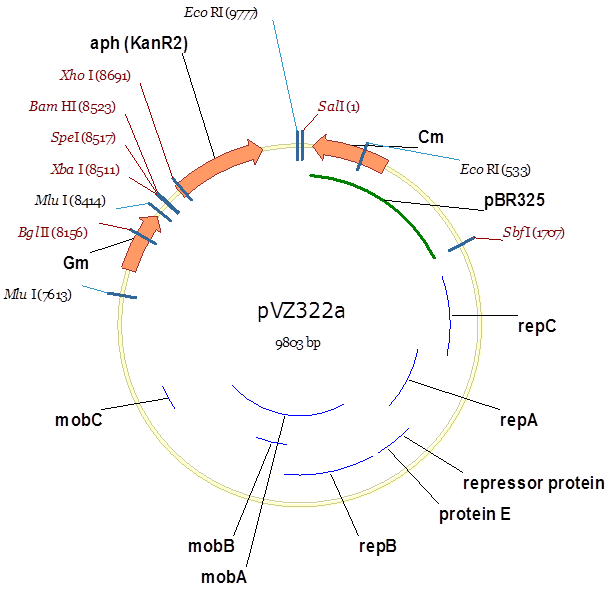


**Additional file 9.** Plasmid maps

Supplement: Supplementary file 9 — Additional file 9. Plasmid maps of pVZ322a and its ethanologenic version #1449 (pVZ322a-corR-PcorT-PDC-dsrA-Prbc*(optRBS)-synADH-oop). The ethanologenic gene cassette contains the PDC gene from Z.mobilis (zmPDC) and the ADH gene from Synechocystis 6803. While expression of PDC is driven by the Co2+ inducible PcorT promoter from Synechocystis 6803, ADH expression is under the control of the genetically engineered Prbc*(optRBS) promoter. corR (sll0794) encodes the transcriptional activator of corT (slr0797). [file 13068_2017_741_MOESM9_ESM.docx]
