# Supplementary material for: Systems analysis of ethanol production in the genetically engineered cyanobacterium Synechococcus sp. PCC 7002
Source: Biotechnol Biofuels. 2017 Mar 6;10:56. doi: 10.1186/s13068-017-0741-0 (PMC5340023; doi:10.1186/s13068-017-0741-0)
Supplement: Supplementary file 10 — Additional file 10. Blue-Native PAGE of protein samples from WT and producer, collected on day 8 and day 22 of the cultivation experiment. Thylakoid membranes equivalent to 8 µg of chlorophyll were separated on precast Novex 4-16% Blue native Gel and stained with Coomassie. Phycobiliproteins are reduced in the producer strain after prolonged cultivation. [file 13068_2017_741_MOESM10_ESM.docx]

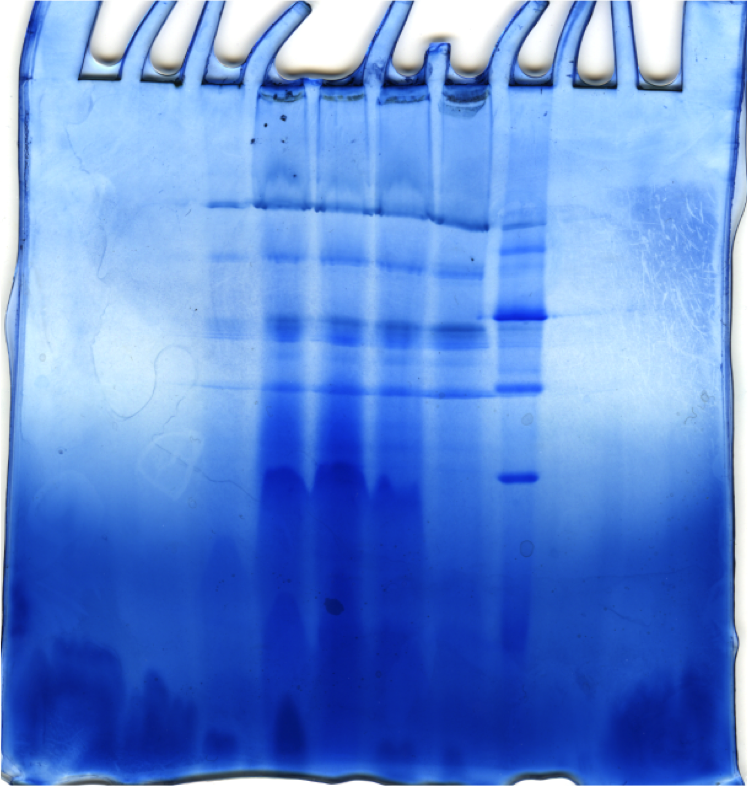


**WT**

**Producer**

8 d 22 d 8 d 22 d

PSI trimer

PSII dimer

PSI/PSII

monomers

Phycobili-

proteins

Coomassie-stained

non-stained


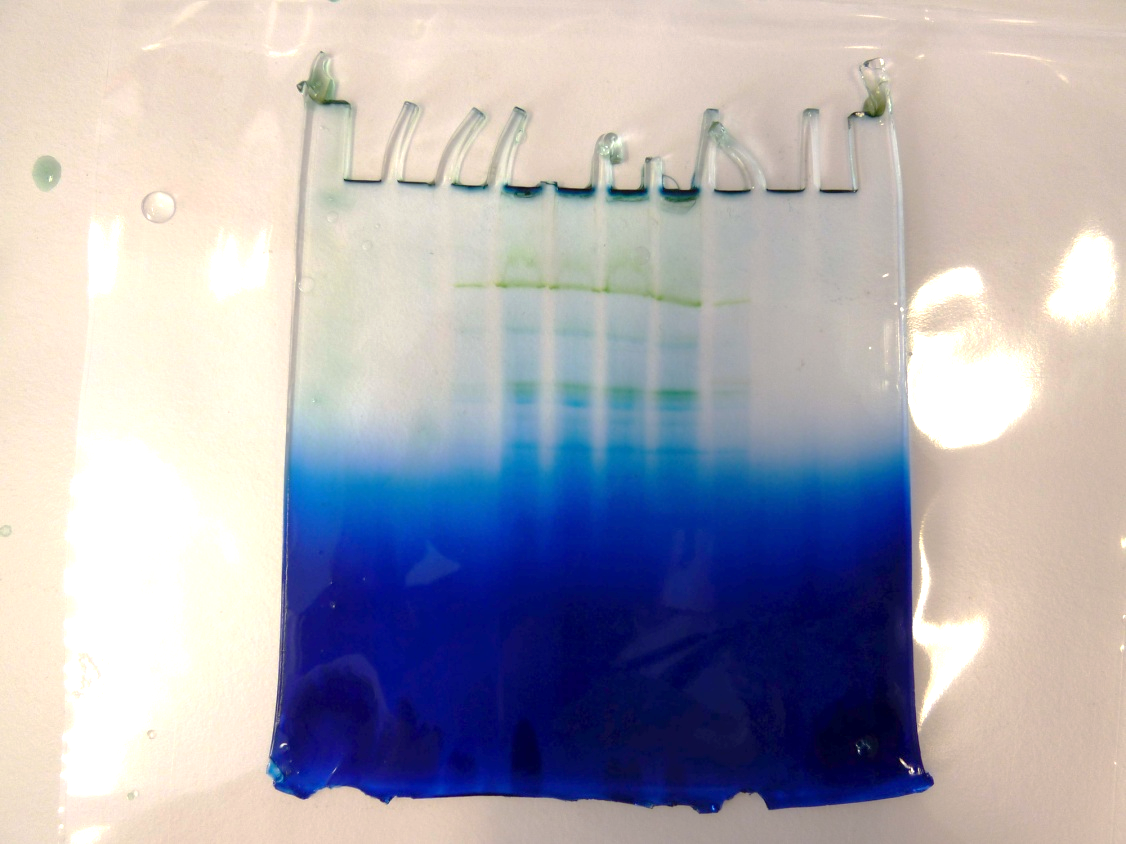


**WT**

**Producer**

8 d 22 d 8 d 22 d

**Additional file 10.** Blue-Native PAGE of WT and producer samples taken on day 8 and day 22 of cultivation.
